# Simulating paired and longitudinal single-cell RNA sequencing data with rescueSim

**DOI:** 10.1093/bioinformatics/btaf442

**Published:** 2025-08-14

**Authors:** Elizabeth A Wynn, Kara J Mould, Brian E Vestal, Camille M Moore

**Affiliations:** Center for Genes, Environment and Health, National Jewish Health, Denver, CO 80206, United States; Department of Medicine, National Jewish Health, Denver, CO 80206, United States; Pulmonary and Critical Care Medicine, University of Colorado Anschutz Medical Campus, Aurora, CO 80045, United States; Center for Genes, Environment and Health, National Jewish Health, Denver, CO 80206, United States; Department of Biostatistics and Informatics, University of Colorado Anschutz Medical Campus, Aurora, CO 80045, United States; Center for Genes, Environment and Health, National Jewish Health, Denver, CO 80206, United States; Department of Biostatistics and Informatics, University of Colorado Anschutz Medical Campus, Aurora, CO 80045, United States

## Abstract

**Motivation:**

As single-cell RNA-sequencing (scRNA-seq) becomes more widely used in transcriptomic research, complex experimental designs, such as paired or longitudinal studies, become increasingly feasible. Paired/longitudinal scRNA-seq enables the study of transcriptomic changes over time within specific cell types, yet guidance on analytical approaches and resources for study planning, such as power analysis, remains limited. Data simulation is a valuable tool for evaluating analysis method performance and informing study design decisions, including sample size selection. Currently, most scRNA-seq simulation methods simulate cells for a single sample, thus ignoring the between-sample and between-subject variability inherent to paired/longitudinal scRNA-seq data.

**Results:**

Here, we introduce rescueSim (REpeated measures Single Cell RNA-seqUEncing data SIMulation), a novel method that simulates paired/longitudinal scRNA-seq data using a gamma-Poisson framework and incorporates additional variability between samples and subjects. We demonstrate our method’s ability to reproduce important data properties and demonstrate its application in study planning.

**Availability and implementation:**

rescueSim is implemented as an R package and is available at https://github.com/ewynn610/rescueSim.

## 1 Introduction

RNA-sequencing (RNA-seq) has revolutionized our understanding of how disease and other biological conditions impact gene expression by simultaneously measuring the expression levels of thousands of genes across the transcriptome (Ozsolak and Milos 2011, Stark *et al.* 2019). Using traditional “bulk” RNA-seq, messenger RNA from all cells in a sample, which may include several distinct cell types, is sequenced together. The resulting expression represents an average across all cells in a sample, and differences in expression between conditions may be due to differences in cell type composition. Single-cell RNA-sequencing (scRNA-seq) technologies have addressed this limitation by enabling the sequencing of RNA from individual cells, eliminating the confounding effects of cell type composition. This has allowed for several novel applications, including the investigation of differential gene expression between biological conditions or disease states for a specific cell type or subpopulation of cells.

**Figure 1.**
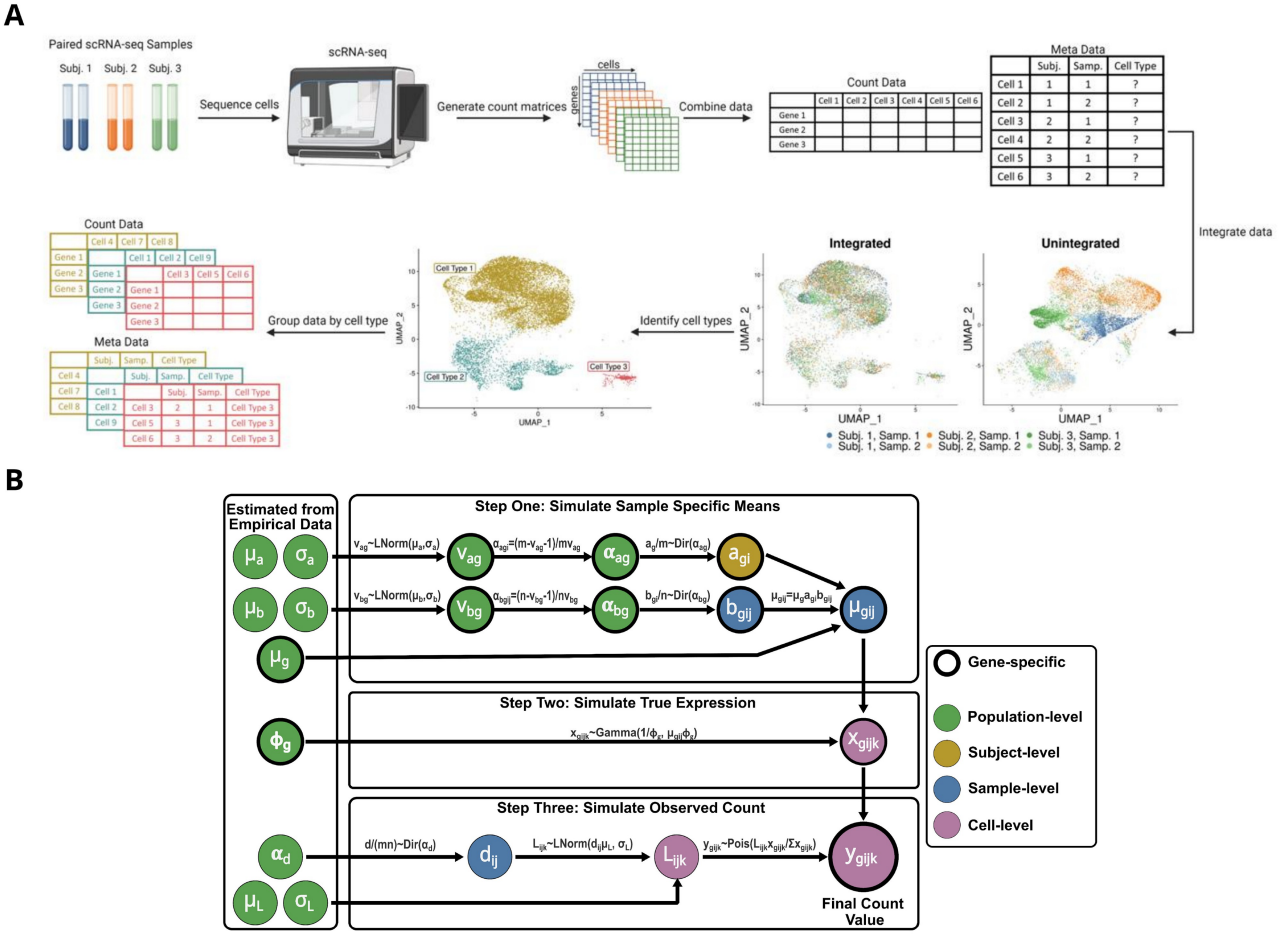
Data/analysis and simulation workflows. (A) Summary of a typical data preparation workflow for paired/longitudinal scRNA-seq data. Paired/longitudinal samples are sequenced, the resulting count matrices are combined and the data are integrated, cell types are identified through clustering, and count data is separated by cell type. (B) Overview of the simulation framework. Parameters and estimates are shown in circles, with the colors indicating whether they are population, subject, sample, or cell-level values. Circles with bold outlines represent parameters estimated at a gene level. The left most values are estimated from empirical data.

With the decreasing cost of scRNA-seq, paired and longitudinal study designs have become feasible, allowing researchers to sequence cells from multiple time points or conditions within the same individual. After processing and integrating the data, cells are typically categorized into specific cell types or subpopulations of cells (Fig. 1A). The repeated measures approach then enables the study of temporal changes in the transcriptome within particular cell types or subpopulations using differential expression (DE) analysis. Although such studies are currently being implemented (Kim *et al.* 2018, Kazer *et al.* 2020, Maynard *et al.* 2020, Ostasov *et al.* 2020), there has yet to be a comprehensive assessment of the best methods to analyze this type of data. Additionally, due to the novelty of paired/longitudinal scRNA-seq studies, there are few resources to guide study design.

The ability to simulate scRNA-seq data for complex study designs could aid in selecting appropriate analysis methods for within-cell type differential expression analysis and inform study design decisions. Simulating data allows researchers to control key data characteristics, enabling them to evaluate and compare the accuracy of statistical methods across various scenarios (Crowell *et al.* 2023). Data simulation can also be useful for planning complex studies where closed-form formulas for power and sample size calculations may not be available (Arnold *et al.* 2011, Su *et al.* 2020). For example, researchers can simulate datasets with different sample sizes and assess power to inform sample size selection.

ScRNA-seq data are typically large and complex, encompassing information from thousands of genes across thousands of cells, making data simulation challenging (Lähnemann *et al.* 2020, Crowell *et al.* 2023). There are many properties that characterize scRNA-seq datasets including the number of cells, cellular library size, average expression levels, and proportion of zero-count observations. One particularly complex aspect of paired/longitudinal scRNA-seq data is its hierarchical structure, where cells are nested within each sample, and multiple samples exist for each subject. This structure introduces both between-sample and between-subject variability, which must be accounted for in analysis and study planning. This variability arises from both biological and technical factors. Biological differences between samples and subjects can cause gene expression in cells from the same sample or from the same subject at different timepoints to be more similar than in cells from different samples or subjects (Zimmerman *et al.* 2021, Junttila *et al.* 2022, Vasaikar *et al.* 2023). Additionally, cells from a single sample are usually processed and sequenced together, which can introduce a technical batch effect (Hicks *et al.* 2018).

Several simulation methods have been developed for scRNA-seq data (Cao *et al.* 2021, Crowell *et al.* 2023). However, most of these methods only simulate data from a single sample, which is generally inadequate for experiments that include multiple biological replicates. Some simulation packages offer options to simulate batch effects (Zappia *et al.* 2017, Zhang *et al.* 2019, Assefa *et al.* 2020, Baruzzo *et al.* 2020). By treating cells from individual samples as batches, these methods can be adapted to simulate multi-sample data. However, the performance of the batch effect modifications to single sample simulation methods has not been fully evaluated in the literature, and the modifications are not always thoroughly described in the associated R package documentation. The only well-developed method for simulating multi-sample scRNA-seq data is the splatPop method (Azodi *et al.* 2021), which simulates counts using a gamma-Poisson framework and introduces sample-level variation by assigning each sample a unique gene-level mean. However, splatPop was not designed to simulate the hierarchical structure inherent to paired or longitudinal designs, where both sample- and subject-level variation are present. While it is possible to adapt splatPop to approximate longitudinal data by treating subjects as populations and using its batch mechanism to simulate multiple samples per subject, this workaround is not straightforward. It requires users to manually specify the distribution of batch effect factors across samples, which may be non-trivial without empirical estimates. Furthermore, splatPop assigns cells to differential expression groups independently of batch (sample), making it difficult to simulate timepoint-specific changes that align with sample identities. These limitations hinder the ability to use splatPop for realistic simulation of longitudinal studies. Currently, there are no methods available that explicitly simulate the hierarchical structure of paired/longitudinal scRNA-seq data, which includes both between-sample and between-subject variability.

In this article, we propose the REpeated measures Single Cell RNA-seqUEncing data simulation (rescueSim) method for simulating paired/longitudinal scRNA-seq data. This method uses a gamma-Poisson framework to simulate counts, incorporating additional variability between cells from different samples and subjects to replicate the hierarchical structure of paired/longitudinal data. rescueSim supports applications focused on within-cell type differential expression by enabling users to simulate gene expression changes across time points or experimental conditions, allowing control over ground truth DE patterns for power calculations or benchmarking studies. We demonstrate that our method can reproduce several key data properties observed in empirical paired/longitudinal scRNA-seq data from various cell types. We also compare rescueSim to splatPop using two approaches: (i) simulating only sample-level correlation, and (ii) simulating subject-level correlation using splatPop’s native population-level correlation structure, combined with its batch mechanism to approximate sample-level variability by splitting each subject into multiple batches. Additionally, we show how rescueSim can be utilized for sample size selection and power analysis in study planning. rescueSim is implemented as an R package, available for installation at https://github.com/ewynn610/rescueSim.

## 2 Simulation framework

Our simulation method generates a paired/longitudinal scRNA-seq dataset for a single cell type or subpopulation of cells, providing gene expression values for *m* subjects, each with *n* samples, and an average of *c* cells per sample, where the user can specify the values for *m*, *n*, and *c*. For multiple cell types of interest, the simulation process can be repeated for each cell type. Empirical data for each cell type is required and can be obtained from pilot data or from a publicly available database such as the Gene Expression Omnibus (Edgar *et al.* 2002). Multi-sample or paired/longitudinal data is not required.

The simulation framework consists of three steps (Fig. 1B). First, we estimate sample-specific mean expression values, with correlation between values for samples from the same subject. Second, using these mean values, we draw “true” expression values for each cell. Finally, we simulate final count values for each cell, incorporating additional variability to represent technical variation. Similar to Baruzzo *et al.* (2020), the *true* gene expression value for gene *g*, subject *i*, sample *j* and cell *k*, denoted as $x_{\mathrm{gijk}}$, is unknown in an empirical dataset, while the *observed* expression value, denoted as $y_{\mathrm{gijk}}$, can be considered the observed count value from an empirical dataset.

### 2.1 Step 1: simulating sample-specific means

#### 2.1.1 Drawing simulated values

Let $\mu_{g}$ be the global mean, representing the average expression of gene *g* across all samples. Due to technical and biological factors, the mean expression of gene *g* for subject *i* and sample *j*, $\mu_{\mathrm{gij}}$, will differ from $\mu_{g}$. To account for between-subject and between-sample variation in mean expression levels, we calculate $\mu_{\mathrm{gij}}=\mu_{g}a_{gi}b_{\mathrm{gij}}$, where $a_{gi}$ and $b_{\mathrm{gij}}$ are multiplicative factors that allow for subject- and sample-specific deviations from the global mean, respectively.

We draw $a_{gi}$ and $b_{\mathrm{gij}}$ from symmetric Dirichlet distributions. Let $a_{g}=\{a_{g1},\ldots a_{gm}\}$ and $b_{\mathrm{gi}}=\{b_{gi1},\ldots ,b_{\mathrm{gin}}\}$. Then


(1) $$\frac{a_{g}}{m}∼\mathrm{Dir}(\alpha_{ag})$$

(2) $$\frac{b_{\mathrm{gi}}}{n}∼\mathrm{Dir}(\alpha_{bg})$$

where $\alpha_{ag}$ and $\alpha_{bg}$ are gene-specific concentration parameters.

To obtain values for $\alpha_{ag}$ and $\alpha_{bg}$, we draw $v_{ag}$ and $v_{bg}$ from log-normal distributions:


(3) $$v_{ag}∼\mathrm{LNorm}(\mu_{a},\sigma_{a}^{2})$$

(4) $$v_{bg}∼\mathrm{LNorm}(\mu_{b},\sigma_{b}^{2})$$

where $v_{ag}$ and $v_{bg}$ are the variances of $a_{g}$ and $b_{gi}$, respectively. The hyper-parameters $\mu_{a}$ and $\mu_{b}$ represent the log-scale mean values for $v_{ag}$ and $v_{bg}$ while $\sigma_{a}^{2}$ and $\sigma_{b}^{2}$ represent the log-scale variances. We use the relationship between the variance and concentration parameter to calculate $\alpha_{ag}$ and $\alpha_{bg}$:


(5) $$\alpha_{\mathrm{agi}}=\frac{m-v_{ag}-1}{m*v_{ag}}$$

(6) $$\alpha_{\mathrm{bgij}}=\frac{n-v_{bg}-1}{n*v_{bg}}$$

#### 2.1.2 Estimating hyper-parameters

We estimate hyper-parameters from empirical data by first normalizing the data using the scran normalization method (Lun *et al.* 2016). For each sample, we then estimate the gene-specific mean value using ${\hat{\mu }}_{\mathrm{gij}}=\frac{\sum_{k}^{c_{ij}}x_{\mathrm{gijk}}}{c_{ij}}$, where $x_{\mathrm{gijk}}$ represents cell-level, gene-specific normalized expression values and $c_{ij}$ is the number of cells from the sample. The overall gene-specific mean, $\mu_{g}$, is then estimated as ${\hat{\mu }}_{g}=\frac{\sum_{i}^{N}\mu_{\mathrm{gij}}}{N}$, where *N* is the total number of samples in the empirical dataset.

The estimation of parameters $\mu_{b}$, $\sigma_{b}$, $\mu_{a}$, and $\sigma_{a}$ requires multi-sample data (for $\mu_{b}$ and $\sigma_{b}$) or paired/longitudinal data (for $\mu_{a}$ and $\sigma_{a}$). If such data is unavailable, we provide plausible values estimated from the datasets described in the Application Dataset section below (Table S1, available as supplementary data at *Bioinformatics* online). When estimating these parameters from empirical data that include samples from different conditions or timepoints, the parameters should be estimated using a set of genes that remain unchanged across conditions/timepoints or after regressing out condition/timepoint effects (Section S1, available as supplementary data at *Bioinformatics* online).

To estimate $\mu_{b}$, $\sigma_{b}$, $\mu_{a}$, and $\sigma_{a}$ from empirical data, we calculate the batch effects $a_{gi}$ and $b_{\mathrm{gij}}$ from the empirical data using the following equations:


(7) $$\mu_{gi}=\mu_{g}a_{gi}$$

(8) $$\mu_{\mathrm{gij}}=\mu_{gi}b_{\mathrm{gij}}$$

(9) $$=\mu_{g}a_{gi}b_{\mathrm{gij}}$$

Once we have generated sample and subject level batch factors for each gene, we take the variance such that:


(10) $$v_{ga}=\mathrm{var}(a_{g})$$

(11) $$v_{gb}=\mathrm{var}(b_{g})$$

Because we calculate $a_{gi}$ and $b_{\mathrm{gij}}$ using sample level means, we would expect some level of variation due to sampling error even if there were no true variation in the sample or subject level means. To account for this, we estimate the gene specific error variance and subtract it from the total variance to get the variance due to between sample/subject variability, $v_{ga}^{*}$ and $v_{ga}^{*}$ (Section S1, available as supplementary data at *Bioinformatics* online).

Finally, to obtain the parameters, $\mu_{b}$, $\sigma_{b}^{2}$, $\mu_{a}$, and $\sigma_{a}^{2}$, we take the mean and variance across genes for $v_{ga}^{*}$ and $v_{ga}^{*}$. Then, using the relationship between the mean/variance and the log mean and variance parameters of the log-normal distribution, we calculate $\mu_{b}$, $\sigma_{b}$, $\mu_{a}$, and $\sigma_{a}$. We found that for genes with a high percentage of 0’s, the estimates for $v_{ga}^{*}$ and $v_{ga}^{*}$ are unstable, so we only use genes with <60% 0 counts to estimate the parameters.

### 2.2 Step 2: simulating true expression values for each cell

#### 2.2.1 Drawing simulated values

Using the sample-specific means estimated in step 1, we draw “true” expression values for each cell from a gamma distribution:


(12) $$x_{\mathrm{gijk}}∼\mathrm{Gamma}(\mathrm{shape}=\frac{1}{\phi_{g}},\mathrm{scale}=\phi_{g}\mu_{\mathrm{gij}})$$

where $\phi_{g}$ is a gene-specific dispersion parameter.

#### 2.2.2 Estimating hyper-parameters

Values of $\phi_{g}$ are calculated from the empirical data using the estimateDisp function in the edgeR package (Robinson and Oshlack 2010). If multi-sample data is provided, the sample with the most cells is used.

### 2.3 Step 3: simulating observed counts for each cell

#### 2.3.1 Drawing simulated values

Before simulating the observed counts, we draw an expected library size for each cell from a log-normal distribution:


(13) $$L_{\mathrm{ijk}}∼\mathrm{LNorm}(d_{ij}\mu_{L},\sigma_{L})$$

where $\mu_{L}$ and $\sigma_{L}$ are the mean and standard deviation of the log-library sizes and $d_{ij}$ is used to simulate sample-specific differences in the average library size due to technical batch effects. Let $d=\{d_{11},\ldots ,d_{mn}\}$. Then we use a symmetric Dirichlet distribution to generate d:


(14) $$\frac{d}{m\times n}∼\mathrm{Dir}(\alpha_{d})$$

where m×n represents the total number of samples being simulated and $\alpha_{d}$ is a vector of identical concentration parameters of length m×n.

Alternatively, users can provide a custom empirical distribution of library sizes from which values are drawn. Within rescueSim, sample-specific shifts in the average value are still applied to maintain realistic heterogeneity across samples.

Using the expected library size values, we scale the simulated true expression values using $x_{\mathrm{gijk}}^{*}=L_{\mathrm{ijk}}\times \frac{x_{\mathrm{gijk}}}{\sum_{g}x_{\mathrm{gijk}}}$ where $x_{\mathrm{gijk}}^{*}$ is the library-adjusted value for $x_{\mathrm{gijk}}$. Then, using these values, we simulate our final observed count values for each cell using a Poisson distribution:


(15) $$y_{\mathrm{gijk}}∼\mathrm{Poisson}(x_{\mathrm{gijk}}^{*})$$

#### 2.3.2 Estimating hyper-parameters

The $\mu_{L}$ and $\sigma_{L}$ parameters are calculated by taking the sample-specific mean and standard deviation of the log library sizes for each sample from the empirical data and then averaging the values across all of the samples.

The concentration parameter $\alpha_{d}$ is estimated by calculating the deviation between the global average log library size and the sample-specific averages, calculating the variance between these deviations, and then generating a parameter estimate using the relationship between the variance and the symmetric Dirichlet concentration parameters previously outlined.

#### 2.3.3 Simulating differential expression

A slight adjustment to step 2 of our framework can be made to simulate differential expression between timepoints. Suppose we have two different timepoints (or biological conditions/disease states), A and B. For the cells in timepoint A, we simulate the true expression value as previously outlined. For timepoint B, we will simulate the true expression values of *N* genes to be differentially expressed from timepoint A, with an expected fold change of $z_{g}$, where $z_{g}>0$. Thus,


(16) $$x_{\mathrm{gijk}}∼\mathrm{Gamma}(\mathrm{shape}=\frac{1}{\phi_{g}},\mathrm{scale}=\phi_{g}\mu_{\mathrm{gij}}z_{g})\;\text{if}\;g\;\text{in}\;1\ldots N$$

(17) $$x_{\mathrm{gijk}}∼\mathrm{Gamma}(\mathrm{shape}=\frac{1}{\phi_{g}},\mathrm{scale}=\phi_{g}\mu_{\mathrm{gij}})\;\text{otherwise}$$

The fold change values ($z_{g}$) are provided by the users. These can take several forms: a single log_2_FC value and a proportion of differentially expressed genes—applied as a ±log_2_FC value between the first and last timepoint with linear interpolation across intermediate timepoints and an even split of up- and downregulation; a vector of logFC values from which individual gene changes are sampled; or a named vector or list of vectors specifying the gene-specific log_2_FC values at each timepoint relative to baseline. The last option allows users to define complex, gene-specific and potentially nonlinear temporal expression patterns.

## 3 Methods

### 3.1 Simulation assessment

#### 3.1.1 Assessment datasets

We assessed our simulation framework using three empirical scRNA-seq datasets. The first consisted of BAL samples from five healthy adult subjects collected at two timepoints, baseline and 4–5 days after administration of lipopolysaccharides (LPS), which were used to induce acute lung inflammation (GEO accessions: GSE151928, GSE300946) (Mould *et al.* 2021). Before simulation, the standard Seurat workflow was used to integrate cells from different samples, cluster cells using a shared nearest neighbor algorithm, and identify markers specific to each cluster for cell type assignment (Stuart *et al.* 2019) (Fig. 1A).

The second dataset contained blood samples from six children with mild/asymptomatic COVID-19, with samples taken at both the acute and convalescent stages of disease (Khoo *et al.* 2023). We used the processed Seurat dataset available on the GEO database (GEO accession: GSE196456) with the cell types annotated using the Azimuth algorithm in Seurat (Hao *et al.* 2021).

The final dataset contained samples taken from bone marrow or peripheral blood of 28 patients with pediatric acute myeloid leukemia (Lambo *et al.* 2023). Samples were taken at three timepoints: diagnosis, remission, and relapse. We used the processed data from the GEO database (GEO accession: GSE235063) including cell type annotations provided by the original authors.

For each dataset, we removed subjects that did not have at least 25 cells at each timepoint, and simulated data for cell types with at least 5 remaining subjects, resulting in three cell types for the Mould *et al.* (2021) dataset, four for the Khoo *et al.* (2023) dataset, and 12 for the Lambo *et al.* (2023) dataset. For each cell type, we removed genes with no counts across all cells and simulated data for the same number of subjects and timepoints as in the empirical dataset. Details for each celltype, including the number of cells and genes in the empirical data, are provided in Table S1, available as supplementary data at *Bioinformatics* online. For each simulation, the number of cells per sample was randomly drawn from a discrete uniform distribution using the 10th and 90th percentile of the cells per sample for each timepoint from the empirical data as parameters. All data were simulated without differential expression.

#### 3.1.2 Comparison with splatPop

We compared the performance of rescueSim with two approaches using splatPop (Azodi *et al.* 2021). First, we used splatPop’s standard framework, which models sample-level correlation but ignores subject-level structure. Second, we adapted splatPop to simulate both sample- and subject-level correlation by treating subjects as the populations and using batch effects to represent repeated samples. While adequate for null simulations, this method is limited when simulating differential expression since splatPop assigns DE status without considering batch, complicating DE modeling between samples. Full details of this implementation are available in Section S2, available as supplementary data at *Bioinformatics* online.

#### 3.1.3 Assessment metrics

We compared the distribution of key metrics between simulated and empirical data, including gene expression mean, variance, proportion of zero counts across genes and cells, and library size. We examined the relationship between metric pairs to assess preservation of bivariate structure.

To evaluate the preservation of sample- and subject-level correlation, we compared tSNE plots, cell mixing scores (Lütge *et al.* 2021), and silhouette widths (Rousseeuw 1987), with the latter two metrics calculated at both the sample and subject levels. Cell mixing scores were computed using the CellMixS package (Lütge *et al.* 2021) and silhouette widths using the cluster package (Maechler 2018). We also compared intra-class correlation (ICC) values for subject and sample, estimated from linear mixed models fit to variance-stabilized data using the lmerSeq package (Vestal *et al.* 2022). These metrics were all calculated using the set of invariant (non-differentially expressed) genes specified prior to simulation (see Section S1, available as supplementary data at *Bioinformatics* online). Because splatPop does not simulate genes that correspond directly to genes in the empirical dataset, we selected genes from the splatPop simulations whose mean expression levels most closely matched those of the empirical invariant genes.

Finally, we quantified agreement between empirical and simulated metrics using Kolmogorov–Smirnov (KS) statistics for univariate and bivariate distributions (Massey 1951, Peacock 1983). Full details of these analyses are provided in Section S3, available as supplementary data at *Bioinformatics* online.

#### 3.1.4 Power analysis

To demonstrate the utility of our method, we performed a hypothetical power analysis for a scRNA-seq study investigating differential expression between timepoints in resident airspace macrophage (RAM) cells. This case example illustrates how power gains might differ depending on whether more subjects, additional timepoints per subject, or increased cell sequencing are used, though the results may vary with other cell types or simulation settings.

We used the RAM empirical data from the Mould *et al.* (2021) dataset to simulate 10 datasets for each of four scenarios. The baseline scenario included five total subjects, two timepoints per subject, and an average of 200 RAM cells per sample. In the remaining scenarios, either the number of subjects, timepoints, or cells was doubled. To generate the data for each scenario, we simulated data with 10 subjects, four timepoints, and 400 cells, then down-sampled the subjects, samples, or cells according to the specifications of each scenario. We also examined how subject- and sample-level variance influenced power by adjusting the $\mu_{a}$ and $\mu_{b}$ parameters in our simulation, which are used to control the mean variance for the subject- and sample-level multiplicative factors. We subtracted or added log(1.5) for both of these parameters to create lower and higher variance conditions, respectively. We randomly drew the number of cells per sample from a uniform distribution with the minimum and maximum values set to ±100 the desired average value. Twenty percent of genes were simulated to have a log_2_ fold change of ±0.35 between the first and final timepoint. In the simulation with four timepoints, we simulated a linear change in expression for differentially expressed genes.

We used the MAST package to assess differential expression in each simulated dataset (Finak *et al.* 2015). This package uses a two-step hurdle model on normalized data to test for differential expression, with a logistic regression model to determine whether the gene is expressed, and a linear model to assess gene expression levels (given the gene is expressed). This model is one of the only scRNA-seq specific methods that allows for multiple random effects, making it suitable for paired/longitudinal data. Before running MAST, we filtered out genes with >90% zero-counts from each simulated dataset and applied the log_2_+1 counts per million transformation suggested in the MAST documentation. We included random intercepts for sample and subject and fixed effects for time and the number of genes detected in a cell, as recommended by Finak *et al.* (2015). Likelihood ratio tests using the combined hurdle method were used to test for differential expression across time. *P*-values were adjusted using the Benjamini–Hochberg method (Benjamini and Hochberg 1995), and we assessed differential expression at the 0.05 threshold. Power was calculated for each dataset. In addition to power, we assessed false discovery rate (FDR) and type one error (T1E) rates across scenarios. The FDR was assessed using Benjamini–Hochberg adjusted *P*-values. The T1E rate was assessed using the set of genes simulated with no differential expression in each simulated dataset and the unadjusted *P*-values.

## 4 Results

### 4.1 Simulation assessment

We evaluated our simulation method across 19 cell types from three empirical datasets, but for brevity, we focus primarily on resident airspace macrophage (RAM) cells from the Mould *et al.* (2020) dataset in the main text. Key metrics for all other cell types are available in Figs S1–S18, available as supplementary data at *Bioinformatics* online.

**Figure 2.**
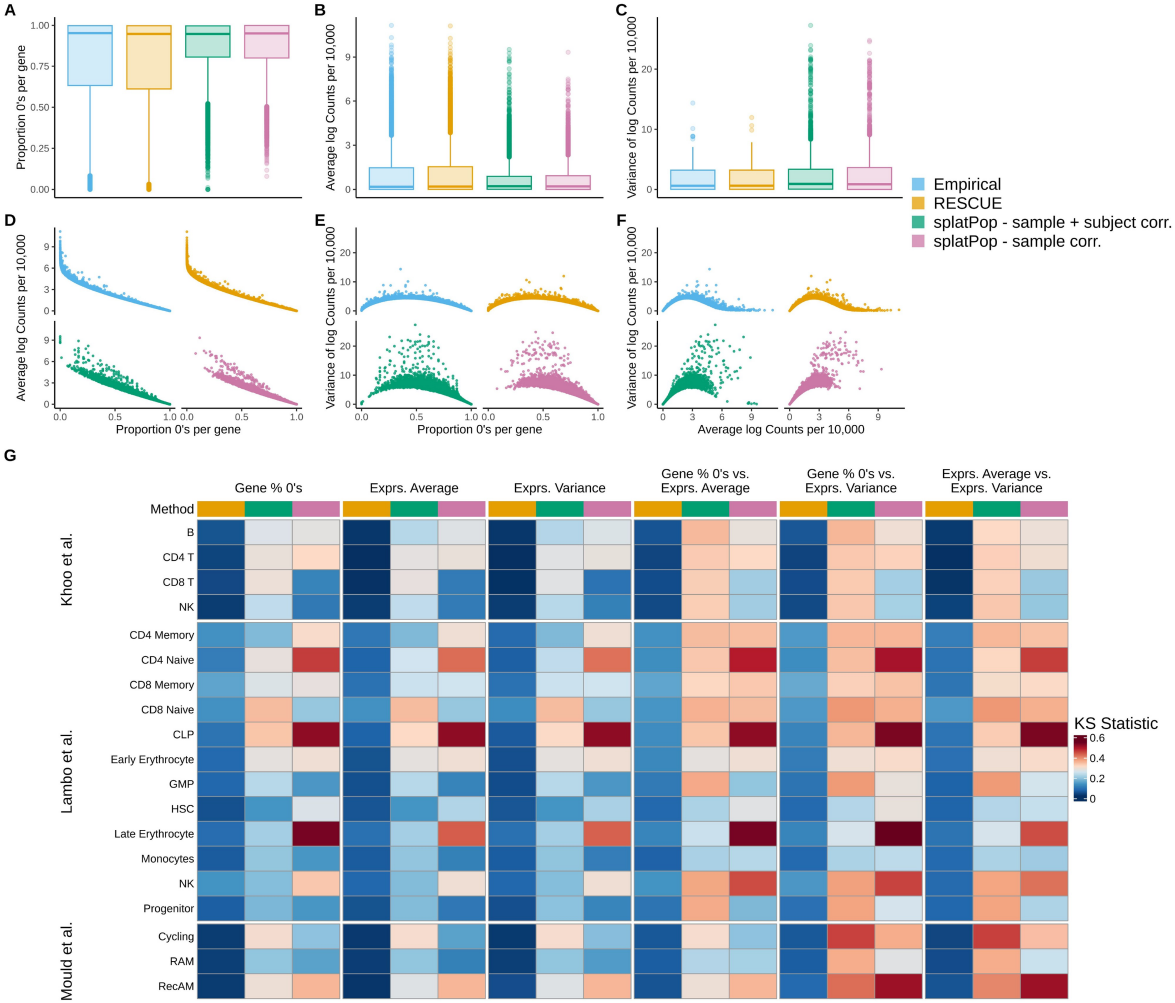
Comparison of gene-level properties between simulated and empirical data. (A–C) Univariate gene-level distributions for RAM cells from the Mould *et al.* (2021) dataset comparing empirical data with simulated data from rescueSim, splatPop with sample-level correlation, and splatPop with subject- and sample-level correlation: (A) proportion of zero counts per gene, (B) average log-transformed counts per million, and (C) variance of log-transformed counts per million. (D–F) Bivariate relationships between gene-level metrics for the same methods and celltype: (D) proportion of zeros versus average log counts per million, (E) proportion of zeros versus variance of log counts per million, and (F) average log counts per million versus variance of log counts per million. (G) Heatmap of Kolmogorov–Smirnov (KS) statistics comparing each simulation method to empirical data across and celltypes in three empirical datasets. Univariate KS statistics were used for individual gene-level metrics and bivariate KS statistics were used for pairs of metrics, with lower values indicating closer similarity to the empirical distribution.

**Figure 3.**
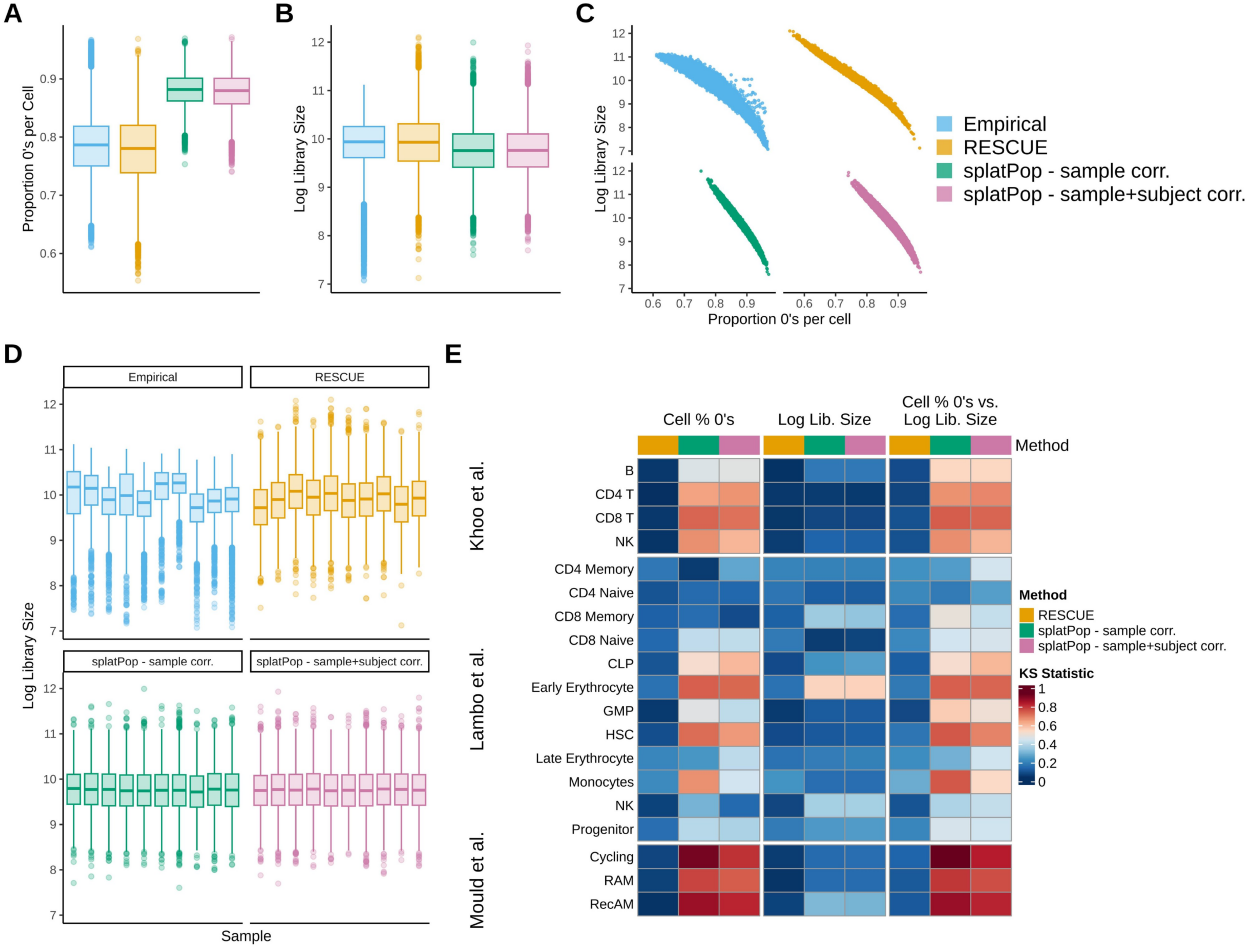
Comparison of cell-level properties between simulated and empirical data. (A–B) Univariate cell-level distributions for RAM cells from the Mould *et al.* (2021) dataset comparing empirical data with simulated data from rescueSim, splatPop with sample-level correlation, and splatPop with subject- and sample-level correlation: (A) proportion of zero counts per cell, and (B) log-transformed library size (total counts per cell). (C) relationship between proportion of zeros per cell and log library size for RAM cells. (D) Distribution of log library sizes across samples for RAM cells. (E) Heatmap of Kolmogorov–Smirnov (KS) statistics comparing each simulation method to empirical data across and celltypes in three empirical datasets. Univariate KS statistics were used for individual cell-level metrics and bivariate KS statistics was used for the bivariate combination of cell-level metrics, with lower values indicating closer similarity to the empirical distribution.

**Figure 4.**
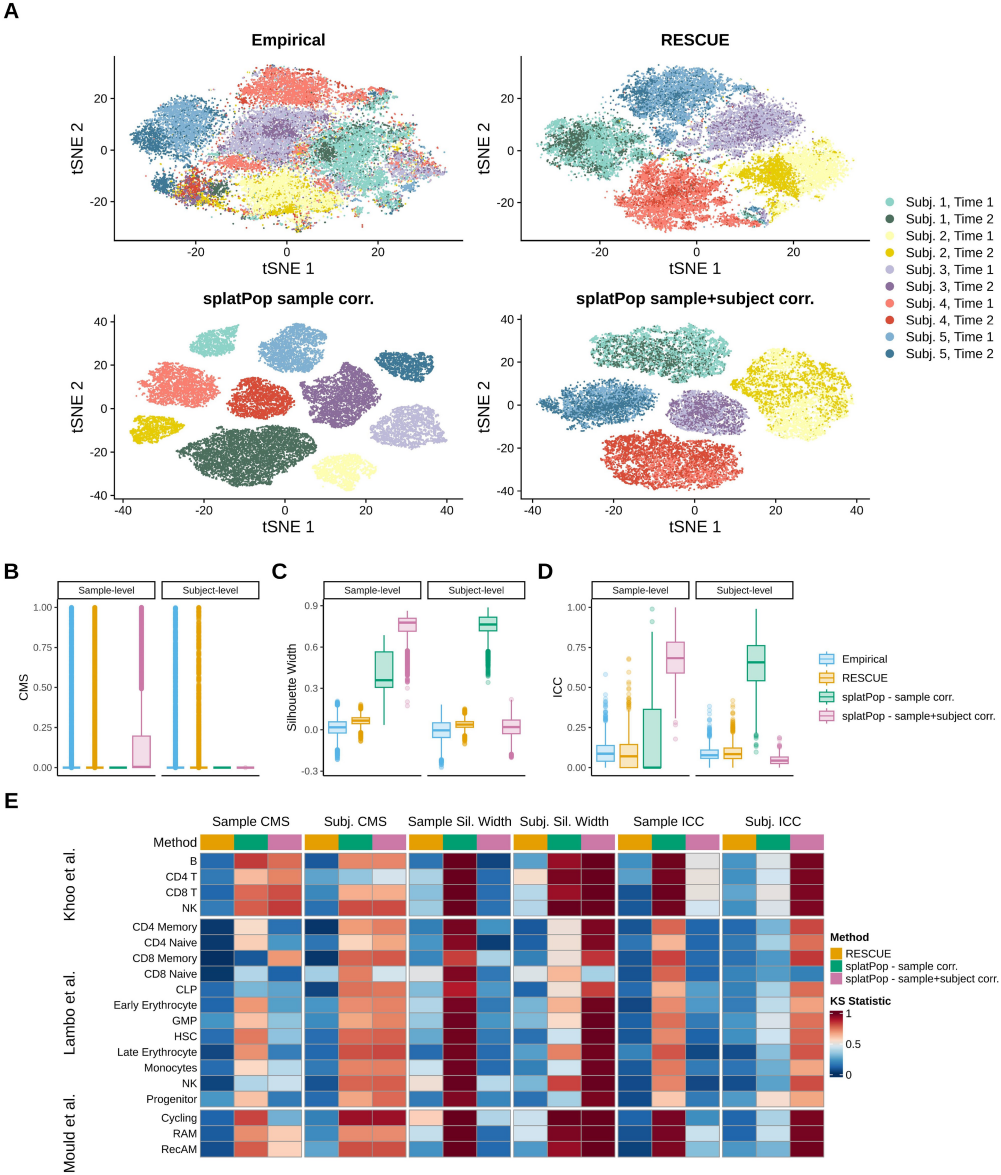
Comparison of sample/subject level variability between simulated and empirical data. (A) t-SNE plots of RAM cells from the Mould *et al.* (2021) dataset colored by subject and shaded by timepoint, demonstrating subject/sample clustering in empirical data, rescueSim simulation, and two splatPop approaches (sample correlation only and sample+subject correlation). (B–D) Distributions of metrics measuring subject- and sample-level correlation and clustering for RAM cells across empirical data and each simulation method: (B) cell mixing score (CMS), (C) silhouette width, and (D) intraclass correlation coefficient (ICC). (E) Heatmap of Kolmogorov–Smirnov (KS) statistics comparing each simulation method to empirical data across and celltypes in three empirical datasets. Lower KS values indicate greater similarity to the empirical structure.

**Figure 5.**
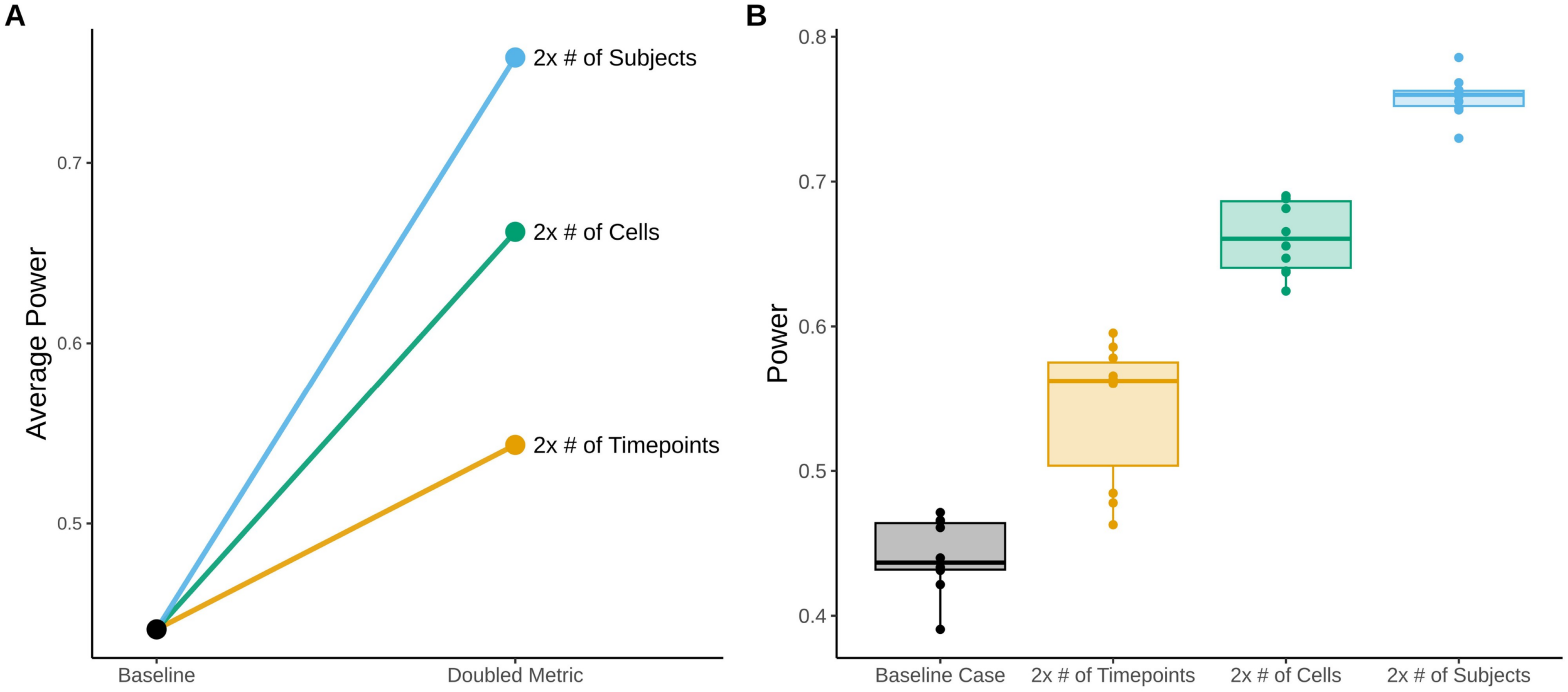
Comparison of power across simulation scenarios in hypothetical scRNA-seq power analysis. (A) Summary plot illustrating the gain in power from doubling the number of subjects, timepoints or cells relative to the baseline scenario. The baseline scenario included 5 subjects, 2 timepoints per subject, and an average of 200 cells per sample. Power is assessed at the 0.05 significance threshold for Benjamini–Hochberg adjusted *P*-values. (B) Boxplot showing the distribution of power across 10 simulations for each scenario at a 0.05 significance threshold.

In RAM cells, rescueSim closely recapitulated the distributions of key gene-level metrics: average and variance of log-normalized counts per million, and percentage of zeros across genes (Fig. 2A–C). In contrast, both splatPop simulation strategies, overestimated data sparsity, underestimated expression levels, and had inflated variance outliers. rescueSim also better preserved the bivariate relationships among these metrics (Fig. 2D–F). Additionally, rescueSim consistently achieved closer alignment with empirical gene-level distributions as evidenced by the uniformly lower KS statistics for rescueSim than for either splatPop approach across all celltypes and gene-level metrics (Fig. 2G).

At the cell level, rescueSim more accurately reflected the distribution of percentage of 0’s per cell in RAM cells, while splatPop overestimated sparsity (Fig. 3A). All methods produced similar mean log-library sizes (Fig. 3B), though simulations lacked some high-end outliers seen in the empirical data, likely due to divergence in the empirical library size distribution from the log-normal model used in the simulations. rescueSim optionally allows for custom library size distributions to be used to address this situation (Fig. S19, available as supplementary data at *Bioinformatics* online). The relationship between log-library size and sparsity was similar across simulation methods (Fig. 3C). Importantly, unlike splatPop, rescueSim also captured sample-specific variation in library size distributions (Fig. 3D). rescueSim also consistently showed lower or comparable KS statistics relative to splatPop for the cell-level metrics across the 19 simulated cell types (Fig. 3E).

Finally, rescueSim better preserved the hierarchical structure of the data. In RAM cells, splatPop (sample-only correlation) produced tightly clustered samples with no subject-level structure, while splatPop (sample + subject correlation) created strong subject-level clustering with overmixed samples (Fig. 4A). rescueSim more closely matched the empirical sample and subject clustering. Quantitative metrics confirmed these results. splatPop (sample-only correlation) showed no sample or subject mixing, while splatPop (subject + sample correlation) showed no subject-level mixing but excessive sample-level mixing (Fig. 4B). For silhouette width splatPop (sample-only correlation) overestimated clustering at both levels, and splatPop (subject + sample correlation) overestimated subject-level silhouette width, but was similar to the empirical data at the sample level (Fig. 4C). rescueSim produced silhouette widths slightly more constrained than the empirical data but overall similar. For ICC, splatPop (sample-only correlation) overestimated the sample-level ICC and showed highly variability in subject-level ICC (Fig. 4D). splatPop (subject + sample correlation) overestimated the subject-level ICC and underestimated the sample-level ICC. rescueSim best matched the empirical ICC distributions at both levels. Across all cell types, rescueSim consistently yielded lower or comparable KS statistics across metrics relative to both splatPop strategies (Fig. 4E).

### 4.2 Power analysis

Using rescueSim, we performed a power analysis to compare gains from recruiting more subjects, adding timepoints, or sequencing more cells. All three strategies increased power (Fig. 5). At a 0.05 significance threshold, the average power for simulations with five subjects, two timepoints per subject, and an average of 200 cells per sample was 0.44. Doubling the number of subjects yielded the largest gain (power = 0.76), followed by increasing cells (power = 0.66) and timepoints (power = 0.54). The ordering remained consistent across additional simulations with lower and higher levels of between subject/sample-level variation (Fig. S20, available as supplementary data at *Bioinformatics* online). As would be expected, power was highest in the low variance scenario. However, gains from adding subjects were most pronounced under high sample/subject variance.

Across all simulations, FDR and T1E rate were slightly elevated with an average FDR of 0.056 and T1E of 0.062 for the baseline scenario (Fig. S21, available as supplementary data at *Bioinformatics* online). Lower-variance simulations showed improved error control, particularly when more cells were added (Fig. S20B and C, available as supplementary data at *Bioinformatics* online).

## 5 Discussion

Single-cell RNA-sequencing is a valuable tool that allows the exploration of gene expression at a cellular level. Paired/longitudinal scRNA-seq experiments enable the study of transcriptional changes over time in individual cell types. However, there is currently no guidance on appropriate analysis methods for these types of studies, nor are there resources to assist with study planning. Simulating data that reproduces the complex structure of paired/longitudinal scRNA-seq data is key to effective analysis method evaluation and can also assist in study design.

In this work we established a simulation method, rescueSim, for paired/longitudinal scRNA-seq data and demonstrated that this method effectively replicates key properties of empirical paired/longitudinal scRNA-seq data across a diverse range of cell types. Compared to two simulation strategies using splatPop, one incorporating only sample-level correlation and the other modeling both sample- and subject-level correlation, rescueSim more accurately recapitulated gene-level distributions, including average expression, expression variance, and sparsity, and better preserved the relationships among these features. At the cell level, rescueSim was able to capture the empirical distribution of library sizes and sparsity, as well as between-sample heterogeneity in library size distributions that splatPop failed to reflect. Notably, rescueSim uniquely retains the hierarchical structure of paired and longitudinal designs, reproducing both sample- and subject-level variation in clustering patterns, cell mixing scores, silhouette widths, and intraclass correlation coefficients. While splatPop can be adapted to introduce subject-level structure using its batch framework, this approach becomes limiting for simulating differential expression, as DE status is assigned independently of batch (sample) identity. In contrast, rescueSim enables simulation of timepoint-specific expression changes, which is important for evaluating methods in longitudinal contexts. Furthermore, across all evaluated metrics and cell types, rescueSim consistently achieved stronger alignment with empirical data than either splatPop strategy, highlighting its utility for generating realistic synthetic data.

We demonstrated the utility of our method by using simulated data to assess how adding subjects, timepoints, or sequencing more cells affects the power to detect differential expression across time. This power analysis served as a case study based on one empirical cell type, so the specific results may vary depending on factors such as the underlying variance structure. Using the MAST package with random effects, we found that increasing the number of subjects consistently yielded the greatest gains in power, followed by adding cells and then timepoints. This pattern held across additional simulations with lower and higher levels of subject- and sample-level variation, consistent with expectations that independent sampling units drive power more than within-sample replication. As anticipated, gains from adding subjects were most pronounced in high-variance settings (Raudenbush and Liu 2000).

We observed modest inflation in FDR and T1E rates in our simulations, a pattern that has also been reported in other studies using MAST with random effects in multi-sample scRNA-seq datasets (Junttila *et al.* 2022, Liu *et al.* 2023, Hafner *et al.* 2024, Hoffman *et al.* 2024). In our simulations, inflation was more pronounced when the number of cells per sample increased. One possible explanation is that between-sample and -subject variability is not fully accounted for in the models, leading to an overestimation of the amount of independent information contributed by each cell, an issue that becomes more evident as cell numbers increase. Notably, error rates were lowest in the low-variance simulation scenario, further supporting the idea that difficulties in modeling subject/sample variation may contribute to the observed inflation. While methods for testing cell-type differential expression in multi-sample scRNA-seq data have been evaluated (Crowell *et al.* 2020, Squair *et al.* 2021, Junttila *et al.* 2022), there has been little to no evaluation of methods in longitudinal settings.

While our simulation framework performed well on 19 different cell types from three empirical datasets, future work applying the framework to additional cell types and study designs would be valuable. Additionally, rescueSim was designed primarily to support benchmarking and power analysis for within-cell type differential expression, and as such, it does not explicitly model complex structures inherent in scRNA-seq data such as correlation structures across cell types. Expanding the framework to capture these features would be a valuable future direction, particularly for evaluating methods focused on cell-cell communication or integrative multi-celltype modeling. Finally, while our method enables users to assess and compare differential expression analysis approaches in paired/longitudinal scRNA-seq studies, we did not conduct such benchmarking in this work. Systematic evaluation of DE methods using rescueSim simulations represents an important next step toward improving analysis strategies for paired and longitudinal single-cell studies.

## Supplementary Material

btaf442_Supplementary_Data

## Data Availability

Baseline BAL data from the Mould *et al.* (2020) dataset are publicly available on the GEO DataSets website under accession number GSE151928, with data from second timepoint after LPS administration available under GSE300946. Data from Khoo *et al.* (2023) can be accessed in GEO under accession GSE196456. Data from Lambo *et al.* (2023) are under accession (GSE235063). The rescueSim R package is currently available on github (https://github.com/ewynn610/rescueSim) and archived on Zenodo (https://doi.org/10.5281/zenodo.15777252). Code used to simulate and summarize data in this manuscript is available on GitHub at https://github.com/ewynn610/rescueSim\_manuscript\_code and archive on Zenodo (https://doi.org/10.5281/zenodo.15777107).
