# Supplementary material for: Simulating paired and longitudinal single-cell RNA sequencing data with rescueSim
Source: Bioinformatics. 2025 Aug 14;41(8):btaf442. doi: 10.1093/bioinformatics/btaf442 (PMC12366488; doi:10.1093/bioinformatics/btaf442)
Supplement: btaf442_Supplementary_Data [file btaf442_supplementary_data.pdf]

Supplemental Material: Simulating Longitudinal Single-cell RNA Sequencing Data with rescueSim

Elizabeth A. Wynn[1], Kara J. Mould[2,3], Brian E. Vestal[1,4], and Camille M. Moore[1,4]

[1]Center for Genes, Environment and Health, National Jewish Health, Denver, CO, USA

[2]Department of Medicine, National Jewish Health, Denver, CO, USA

[3]Department of Pulmonary and Critical Care Medicine, University of Colorado Anschutz Medical Campus, Aurora, CO, USA

[4]Department of Biostatistics and Informatics, University of Colorado Anschutz Medical Campus, Aurora, CO, USA

S1 Estimating Batch Effect Hyper-Parameters

To estimate the batch variance hyper-parameters μ_b, σ_b, μ_a, and σ_a from empirical data, we first generate a set of genes that is roughly invariant across timepoints or conditions. To identify this set of genes, we aggregate counts for each sample for each gene, and then use the `edgeR` package to fit models for each gene with the condition/timepoint variable as a fixed effect. If the empirical data are longitudinal, subject is also included as a fixed effect. Genes with $\log_2$-fold changes between -0.1 and 0.1 are used to estimate parameters.

Because we calculate a_{gi} and b_{gij} using sample level means, we would expect some level of variation due to sampling error even if there were no true variation in the sample or subject level means. Using central limit theorem we can approximate the degree of variation due to sampling error as:

$$v_{gerror} = \frac{\phi_g * x_g^2 + x_g}{n * x_g^2} \tag{1}$$

where ϕ_g is the dispersion for gene g, x_g is the global mean expression, and n is the average number of cells per sample/subject. We subtract the gene specific error variance from the total variance to get the variance due to between sample/subject variability which we will refer to as v^*_{ga}, v^*_{ga}.

Last, to get the parameters, μ_b, σ^2_b, μ_a, and σ^2_a, we take the mean and variance across genes for v^*_{ga} and

v^*_{ga}. Then, using the relationship between the mean/variance and the log mean and variance parameters of the log-normal distribution, we calculate μ_b, σ_b, μ_a, and σ_a. We found that for genes with a high percentage of 0's, the estimates for v^*_{ga} and v^*_{ga} are unstable, so we only use genes with $< 60\%$ 0 counts to estimate the parameters.

S2 splatPop Simulation Methods

We simulated data using splatPop in two ways to compare with rescueSim. First we used splatPop's standard approach, modeling sample-level correlation while treating samples as independent. Second, we adapted splatPop to simulate both subject- and sample-level correlation. In this adaptation, each subject was treated as a population, and batch effects were used to represent repeated measures by simulating multiple batches nested within each subject.

For both simulation strategies, we estimated splatPop parameters using pseudo-bulk means aggregated at either the sample or subject level, alongside counts from the largest empirical sample. For the population coefficient of variation, if estimation failed due to too many CV bins, we reduced the number of bins (starting at 10) until estimation succeeded.

The number of cells per sample was simulated using a gamma distribution as implemented in splatPop. We fitted gamma parameters to the empirical distribution of cell counts per sample using `fitdistrplus` Delignette-Muller and Dutang [2015].

In the subject/sample structure simulation, subjects were represented as populations in splatPop. Repeated measures were introduced by simulating batches, where each batch represented a sample from that subject. In splatPop, batch effects are introduced by multiplying expression mean by batch-specific factors drawn from a log-normal distribution. the user must set two parameters `batch.facLoc`, which controls the center of the distribution on the log scale, and `batch.facScale`, which controls the variation of these multiplicative factors.

We set `batch.facLoc = 0`, so that batch factors were centered at $exp(0) = 1$, meaning no average shift between batches. We set `batch.facScale` to match the expected standard deviation of the multiplicative factors in rescueSim. Specifically, we calculated:

$$\texttt{batch.facScale} = \exp\left(\frac{\texttt{sampleFacVarMean}}{2} + \frac{\texttt{sampleFacVarSD}^2}{8}\right)$$

where `sampleFacVarMean` and `sampleFacVarSD` are parameters estimated in rescueSim and reflect the parameters of the log-normal distribution used to draw the variance for the multiplicative factors. This

ensured that the batch multiplicative factors in splatPop simulations had the same expected SD as those drawn in rescueSim. It is important to note that in rescueSim, the variance for drawing the sample-level multiplicative factors is gene-specific, whereas in splatPop the same variance is used to draw batch factors for all genes within a batch.

S3 Assessment Metrics

S3.1 tSNE embeddings

We generated tSNE plots on simulated and empirical data for visual comparison of clustering by sample and subject. Embedding were based on the first 30 principal components from PCA. Plots were generated using the `Seurat` workflow [Hao et al., 2024].

S3.2 Cell Mixing Score

We calculated sample- and subject-level cell mixing score using the `CellMixS` package [Lütge et al., 2021]. We used the density based neighborhood approach with k equal to the larger of 10 or the median number of cells per sample/subject in the empirical data and a k_min of 10.

S3.3 Silhouette Width

We calculate silhouette widths using the `cluster` package [Martin Maechler; Peter Rousseeuw; Anja Struyf; Mia Hubert; Kurt Hornik, 2023]. Distance were computed using Euclidean distances on PCA embeddings. We assessed silhouette width for both sample and subject labels.

S3.4 Intra-class correlation (ICC)

WE evaluated ICC at both sample and subject levels. Data were variance stabilized using `sctransform` [Hafemeister and Satija, 2019]. We fit LMMs on the 800 genes with the highest mean normalized counts using `lmerSeq` [Vestal et al., 2022], with random intercepts for sample and subject. ICC values were calculated as the proportion of variance attributable to each random effect relative to the total variance.

S3.5 Kolmogorov-Smirnov (KS) statistics

We used KS statistics to quantify difference between empirical and simulated distributions. Univariate statistics were computed using `ks.test` and bivariate statistics were computed using the `Peacock.test` package.

S4 Supplemental Tables and Figures

Table S1: Summary of simulated cell types and batch level hyper-parameters for each cell type

Cell Type	Dataset	# of Cells	# of Genes	μ_a	σ_a	μ_b	σ_b
B	Khoo et al., 2023	38,359	22,927	-5.75	1.75	-7.00	1.45
CD4 T	Khoo et al., 2023	102,262	23,171	-5.75	1.74	-7.01	1.50
CD8 T	Khoo et al., 2023	63,991	23,158	-5.55	1.57	-6.35	0.90
NK	Khoo et al., 2023	26,871	22,362	-5.50	1.54	-6.35	1.00
CD4 Memory	Lambo et al., 2023	8,103	20,427	-3.70	1.11	-3.37	0.64
CD4 Naive	Lambo et al., 2023	27,608	21,956	-3.45	0.67	-3.72	0.76
CD8 Memory	Lambo et al., 2023	2,824	17,430	-3.71	0.96	-3.57	0.78
CD8 Naive	Lambo et al., 2023	1,849	16,389	-3.63	1.23	-3.96	0.68
CLP	Lambo et al., 2023	3,697	20,653	-3.21	0.74	-3.17	0.72
Early Erythrocyte	Lambo et al., 2023	9,053	22,208	-3.17	0.93	-3.36	0.63
GMP	Lambo et al., 2023	23,804	24,747	-3.01	0.80	-3.10	0.61
HSC	Lambo et al., 2023	22,762	24,346	-2.96	0.71	-3.20	0.63
Late Erythrocyte	Lambo et al., 2023	3,941	18,621	-2.91	0.71	-3.55	0.59
Monocytes	Lambo et al., 2023	29,787	23,933	-3.26	0.59	-3.09	0.54
NK	Lambo et al., 2023	4,092	18,180	-3.66	0.77	-3.72	0.88
Progenitor	Lambo et al., 2023	51,026	25,856	-2.76	0.59	-2.88	0.50
Cycling	Mould et al., 2020	1,540	18,196	-4.68	1.13	-5.25	0.99
RAM	Mould et al., 2020	26,998	21,364	-4.84	1.14	-5.69	0.87
RecAM	Mould et al., 2020	9,041	19,410	-5.06	1.20	-5.57	0.90

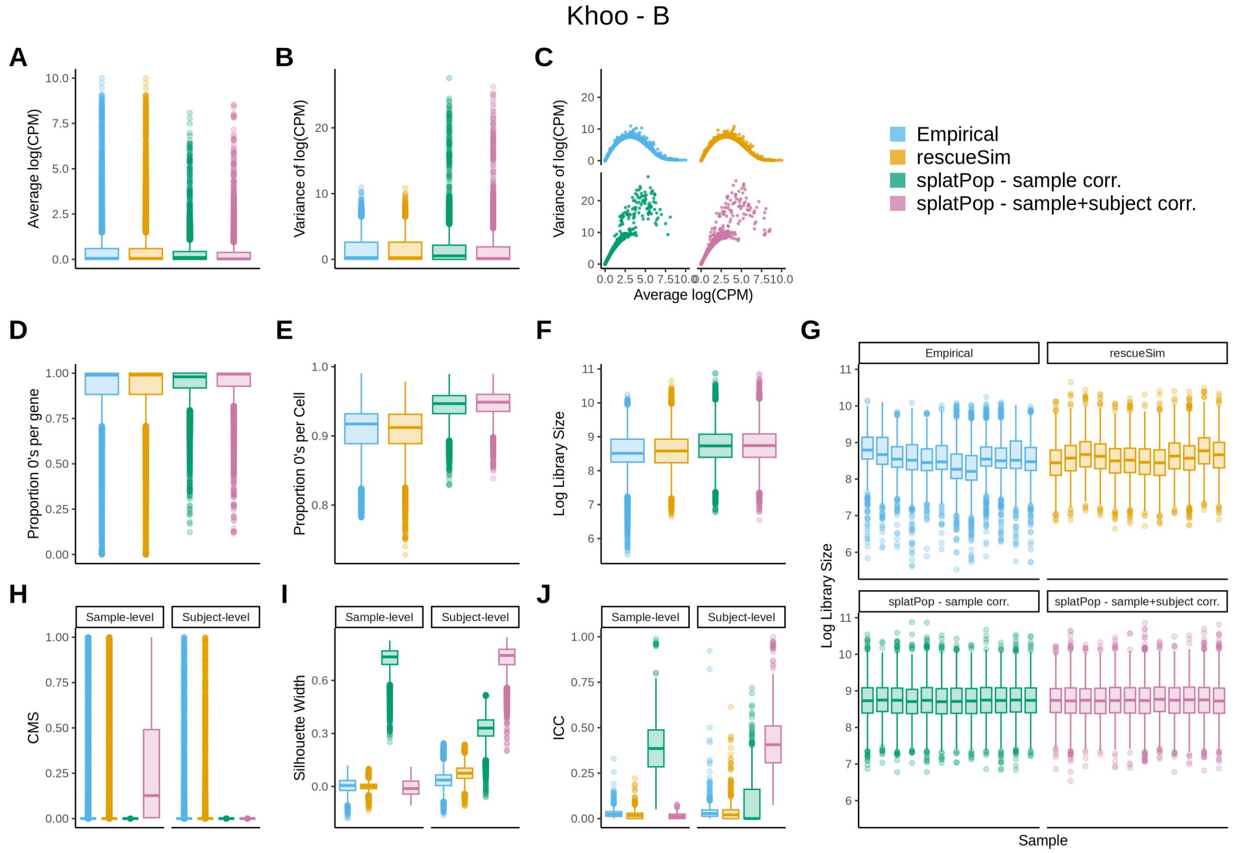

Figure S1: Comparison of key data metrics between simulated and empirical B-cell data (Khoo et al., 2023). (A) Average log-transformed counts per million (CPM), (B) variance of log-transformed counts per million, (C) average log counts per million vs. variance of log counts per million, (D) proportion of zero counts per gene, (E) proportion of zero counts per cell (F) log-transformed library size (total counts per cell), (G) distribution of log library sizes across samples for RAM cells, (H) Cell mixing score (CMS), (I) silhouette width, and (J) intraclass correlation coefficient (ICC).

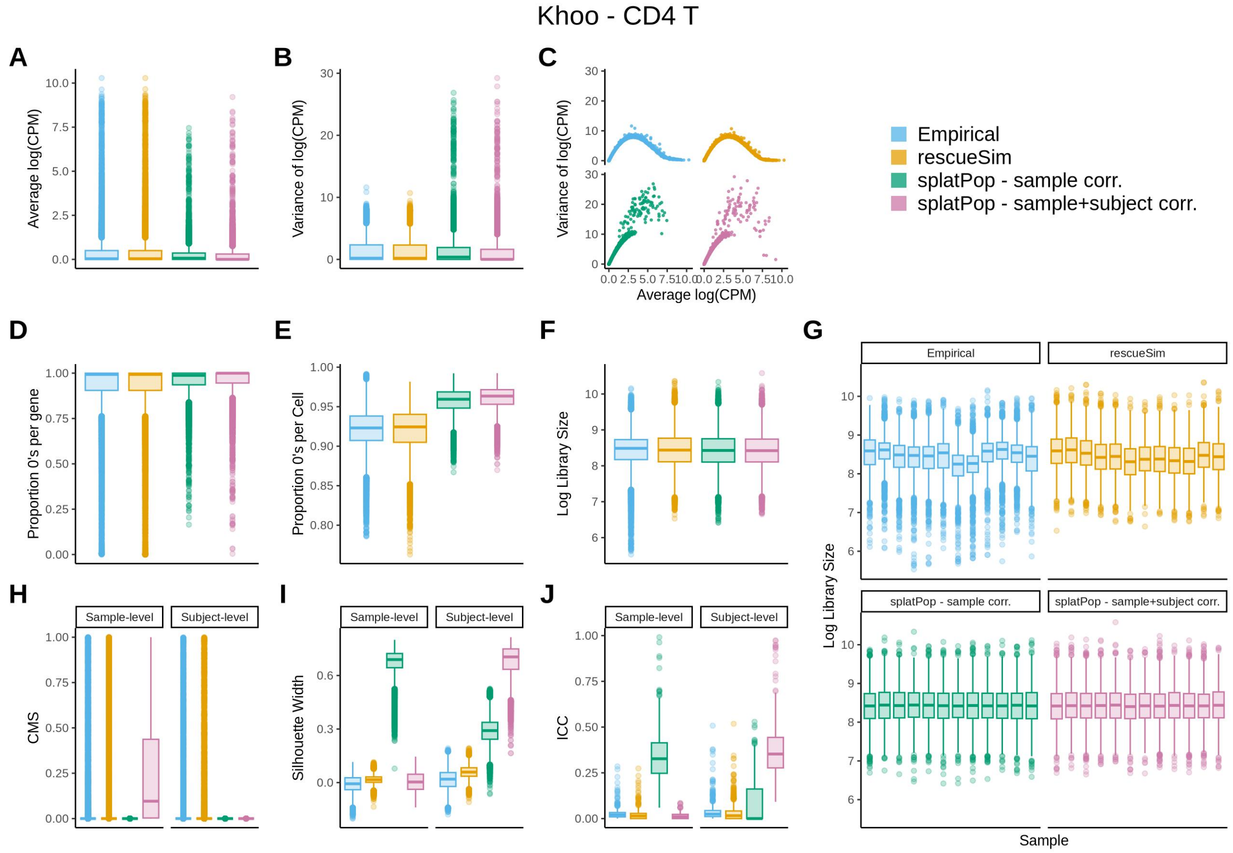

Figure S2: Comparison of key data metrics between simulated and empirical CD4 T-cell data (Khoo et al., 2023). (A) Average log-transformed counts per million (CPM), (B) variance of log-transformed counts per million, (C) average log counts per million vs. variance of log counts per million, (D) proportion of zero counts per gene, (E) proportion of zero counts per cell (F) log-transformed library size (total counts per cell), (G) distribution of log library sizes across samples for RAM cells, (H) Cell mixing score (CMS), (I) silhouette width, and (J) intraclass correlation coefficient (ICC).

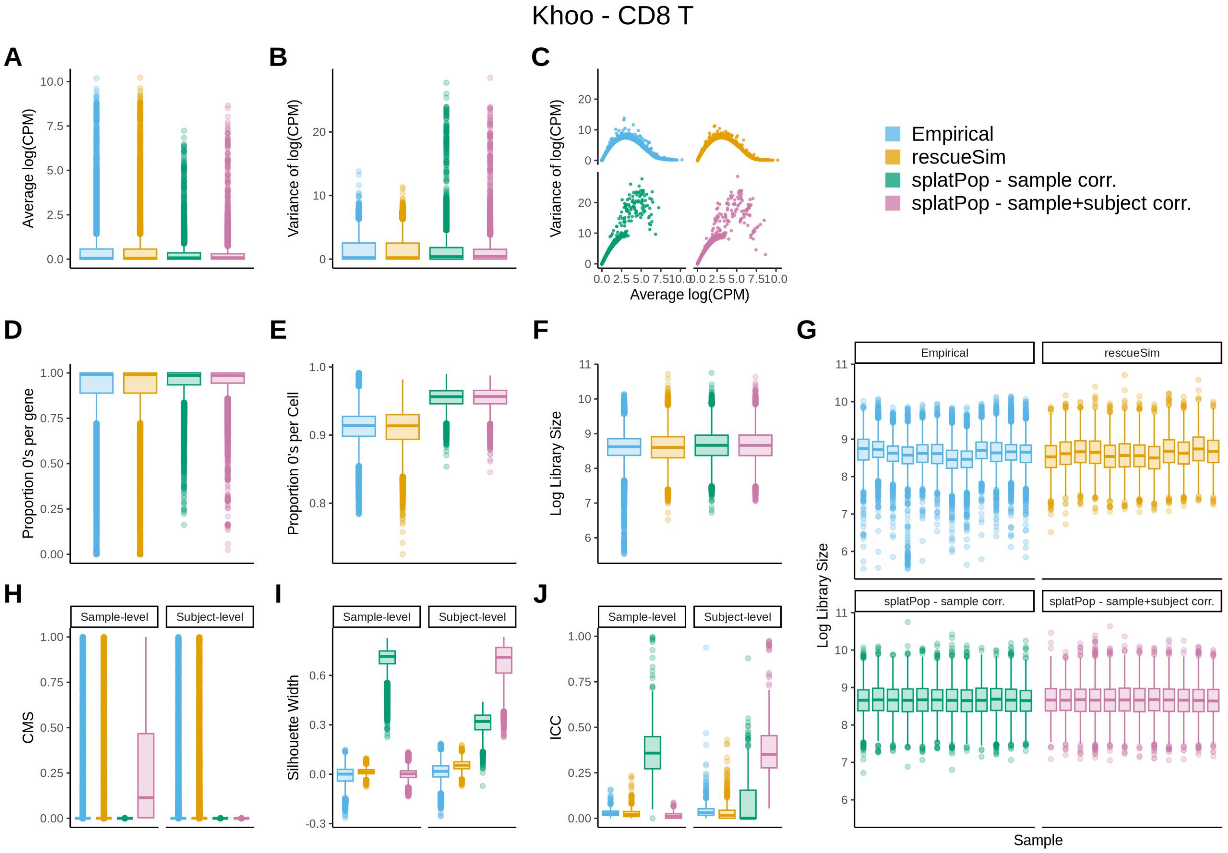

Figure S3: Comparison of key data metrics between simulated and empirical CD8 T-cell data (Khoo et al., 2023). (A) Average log-transformed counts per million (CPM), (B) variance of log-transformed counts per million, (C) average log counts per million vs. variance of log counts per million, (D) proportion of zero counts per gene, (E) proportion of zero counts per cell (F) log-transformed library size (total counts per cell), (G) distribution of log library sizes across samples for RAM cells, (H) Cell mixing score (CMS), (I) silhouette width, and (J) intraclass correlation coefficient (ICC).

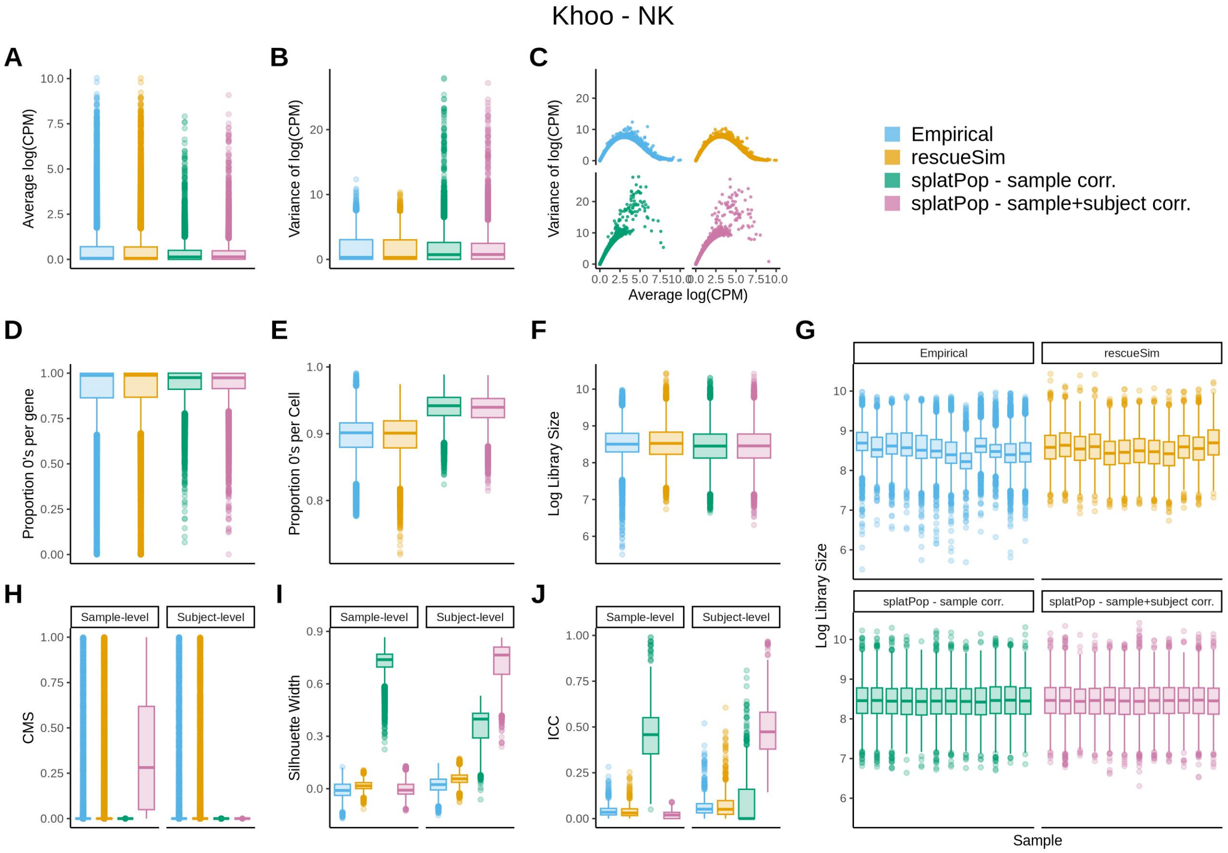

Figure S4: Comparison of key data metrics between simulated and empirical NK cell data (Khoo et al., 2023). (A) Average log-transformed counts per million (CPM), (B) variance of log-transformed counts per million, (C) average log counts per million vs. variance of log counts per million, (D) proportion of zero counts per gene, (E) proportion of zero counts per cell (F) log-transformed library size (total counts per cell), (G) distribution of log library sizes across samples for RAM cells, (H) Cell mixing score (CMS), (I) silhouette width, and (J) intraclass correlation coefficient (ICC).

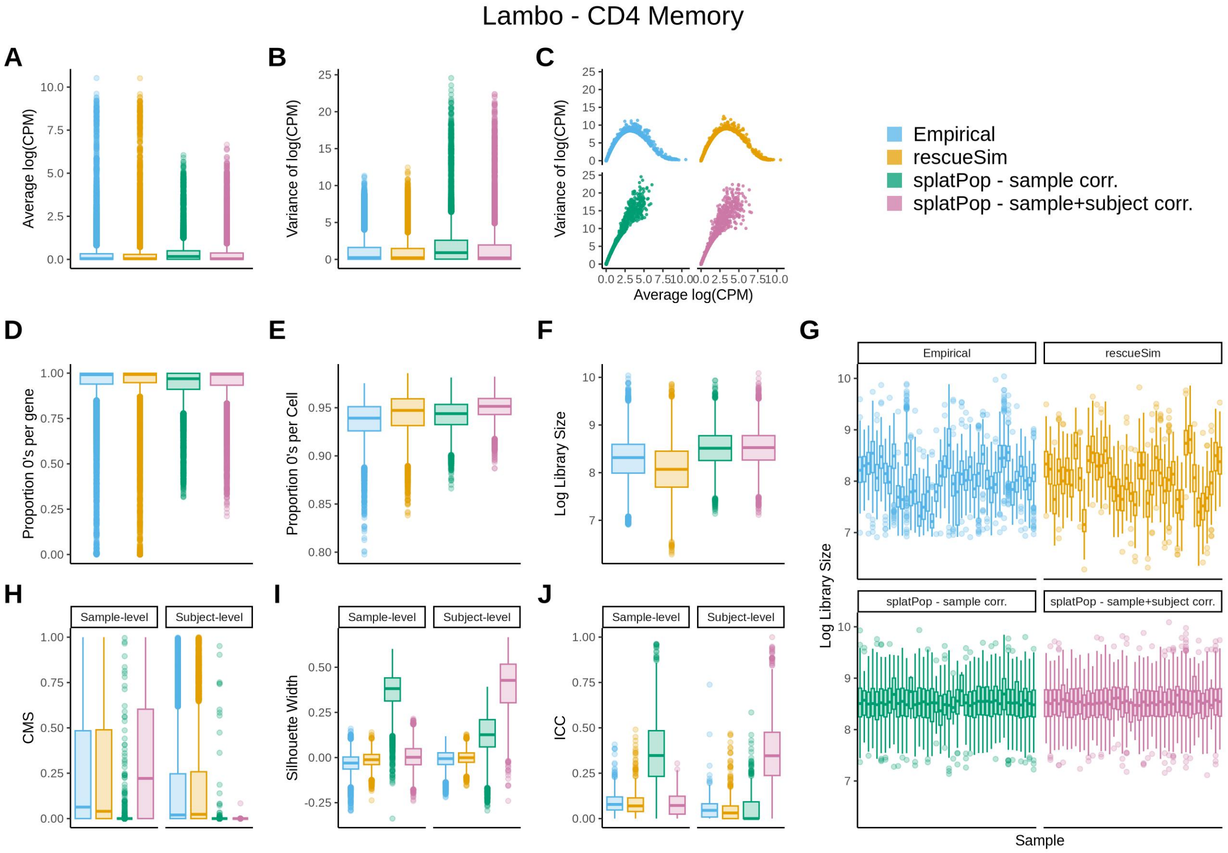

Figure S5: Comparison of key data metrics between simulated and empirical CD4 memory cell data (Lambo et al., 2023). (A) Average log-transformed counts per million (CPM), (B) variance of log-transformed counts per million, (C) average log counts per million vs. variance of log counts per million, (D) proportion of zero counts per gene, (E) proportion of zero counts per cell (F) log-transformed library size (total counts per cell), (G) distribution of log library sizes across samples for RAM cells, (H) Cell mixing score (CMS), (I) silhouette width, and (J) intraclass correlation coefficient (ICC).

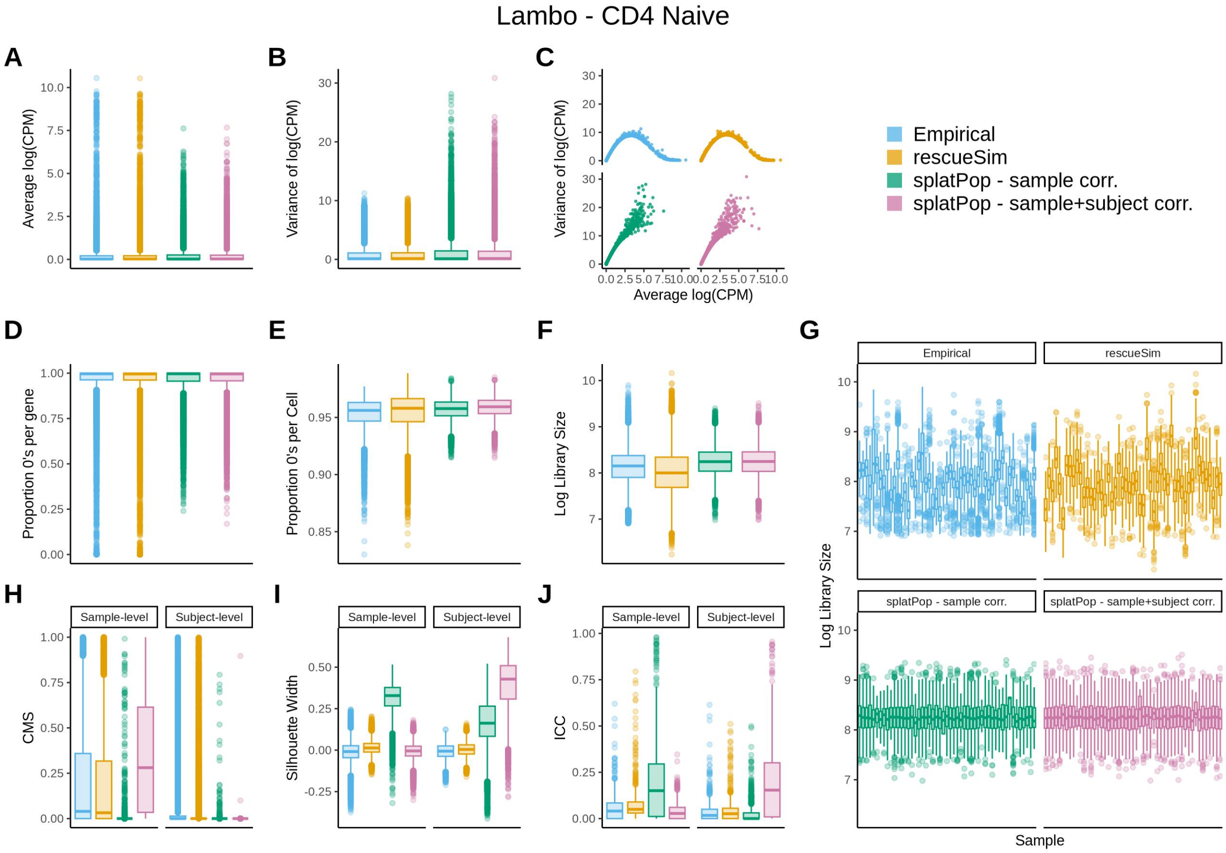

Figure S6: Comparison of key data metrics between simulated and empirical CD4 naive cell data (Lambo et al., 2023). (A) Average log-transformed counts per million (CPM), (B) variance of log-transformed counts per million, (C) average log counts per million vs. variance of log counts per million, (D) proportion of zero counts per gene, (E) proportion of zero counts per cell (F) log-transformed library size (total counts per cell), (G) distribution of log library sizes across samples for RAM cells, (H) Cell mixing score (CMS), (I) silhouette width, and (J) intraclass correlation coefficient (ICC).

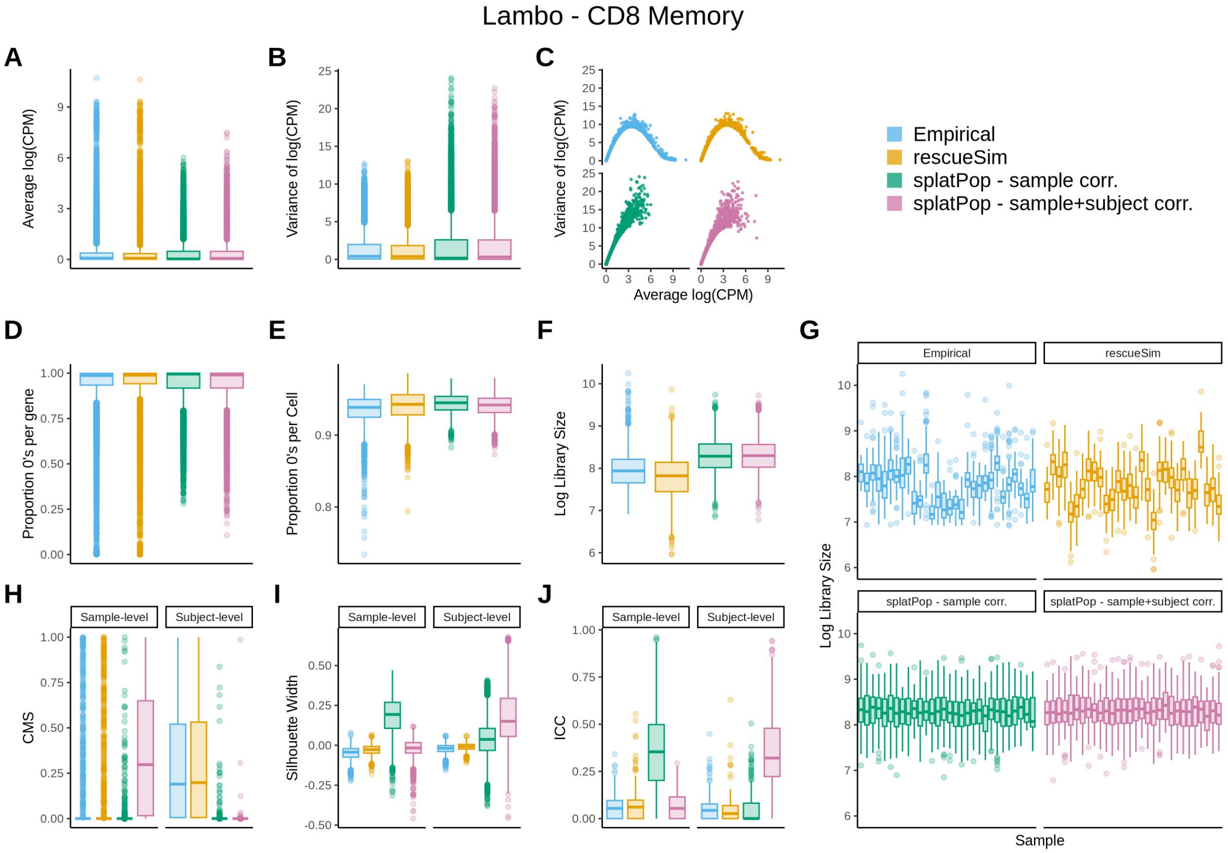

Figure S7: Comparison of key data metrics between simulated and empirical CD8 memory cell data (Lambo et al., 2023). (A) Average log-transformed counts per million (CPM), (B) variance of log-transformed counts per million, (C) average log counts per million vs. variance of log counts per million, (D) proportion of zero counts per gene, (E) proportion of zero counts per cell (F) log-transformed library size (total counts per cell), (G) distribution of log library sizes across samples for RAM cells, (H) Cell mixing score (CMS), (I) silhouette width, and (J) intraclass correlation coefficient (ICC).

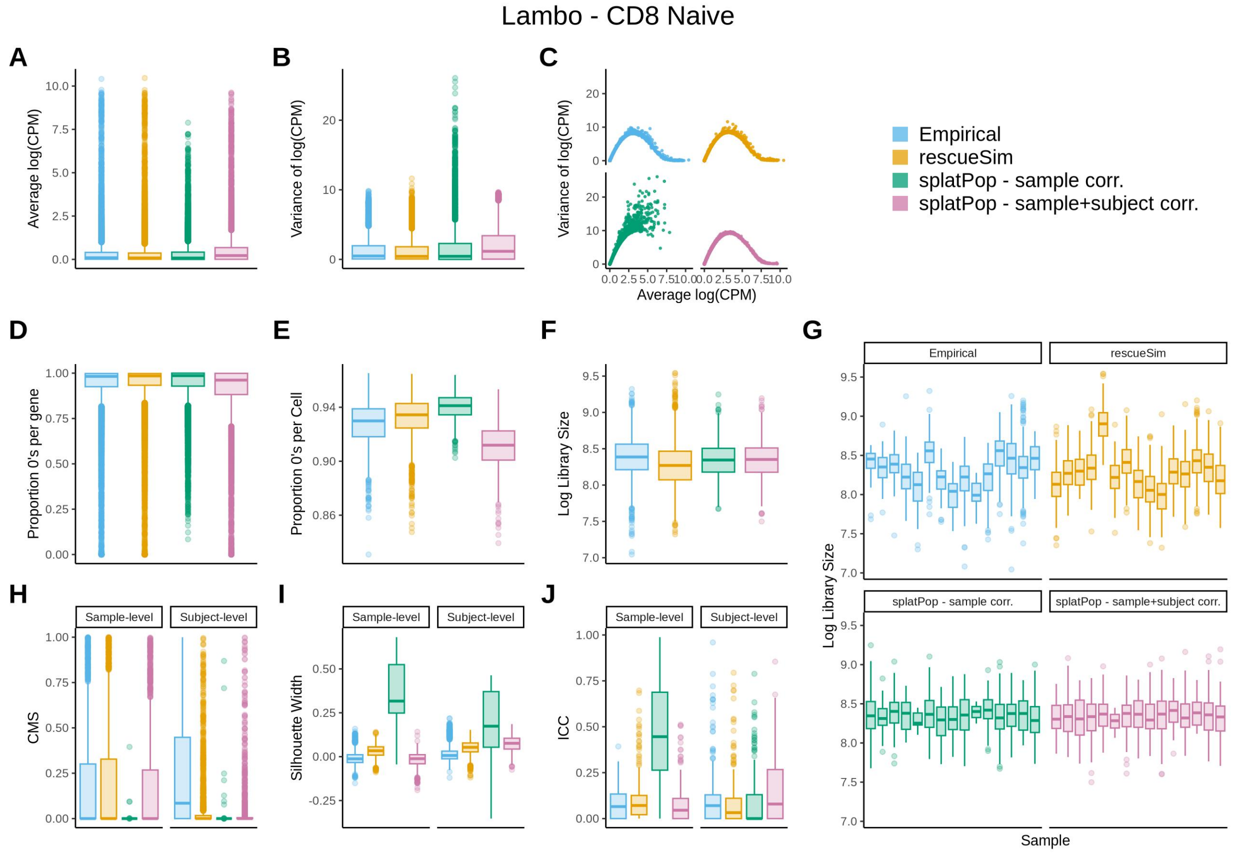

Figure S8: Comparison of key data metrics between simulated and empirical CD8 naive cell data (Lambo et al., 2023). (A) Average log-transformed counts per million (CPM), (B) variance of log-transformed counts per million, (C) average log counts per million vs. variance of log counts per million, (D) proportion of zero counts per gene, (E) proportion of zero counts per cell (F) log-transformed library size (total counts per cell), (G) distribution of log library sizes across samples for RAM cells, (H) Cell mixing score (CMS), (I) silhouette width, and (J) intraclass correlation coefficient (ICC).

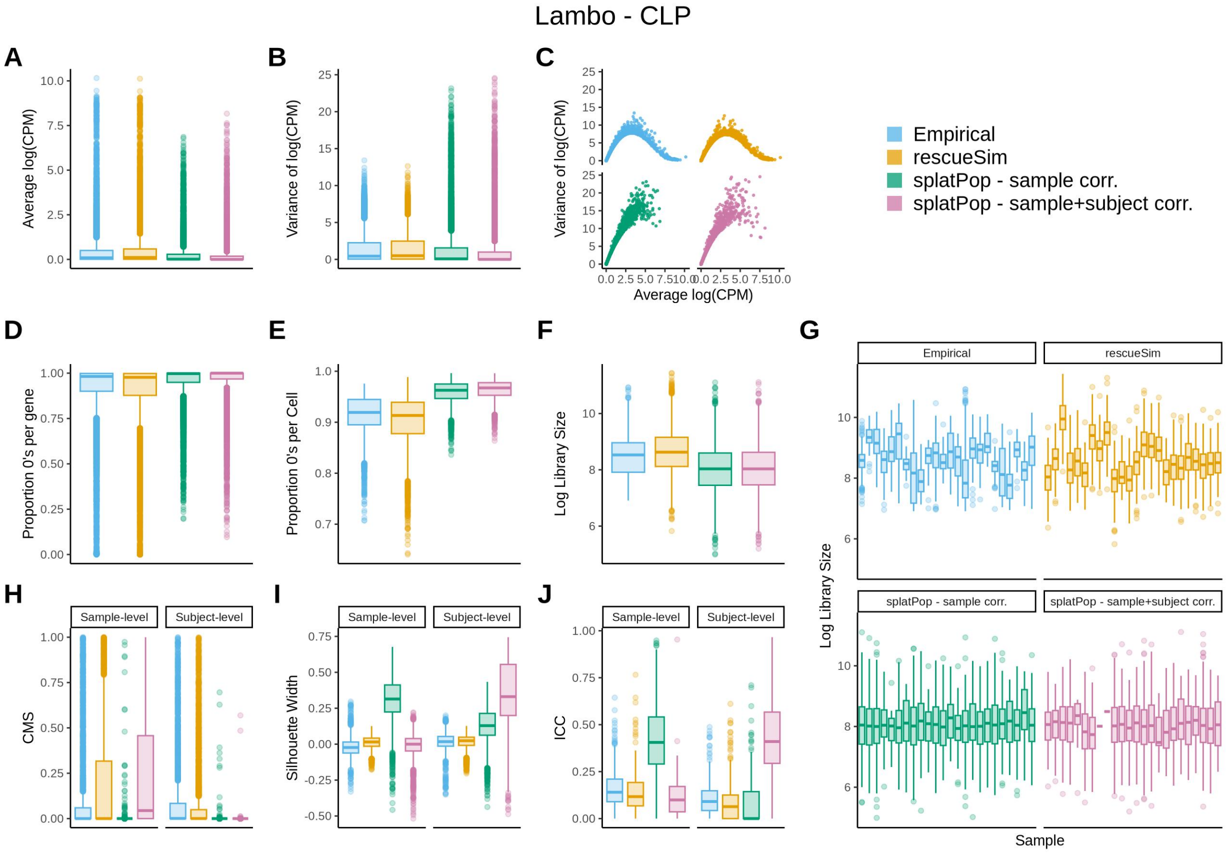

Figure S9: Comparison of key data metrics between simulated and empirical CLP cell data (Lambo et al., 2023). (A) Average log-transformed counts per million (CPM), (B) variance of log-transformed counts per million, (C) average log counts per million vs. variance of log counts per million, (D) proportion of zero counts per gene, (E) proportion of zero counts per cell (F) log-transformed library size (total counts per cell), (G) distribution of log library sizes across samples for RAM cells, (H) Cell mixing score (CMS), (I) silhouette width, and (J) intraclass correlation coefficient (ICC).

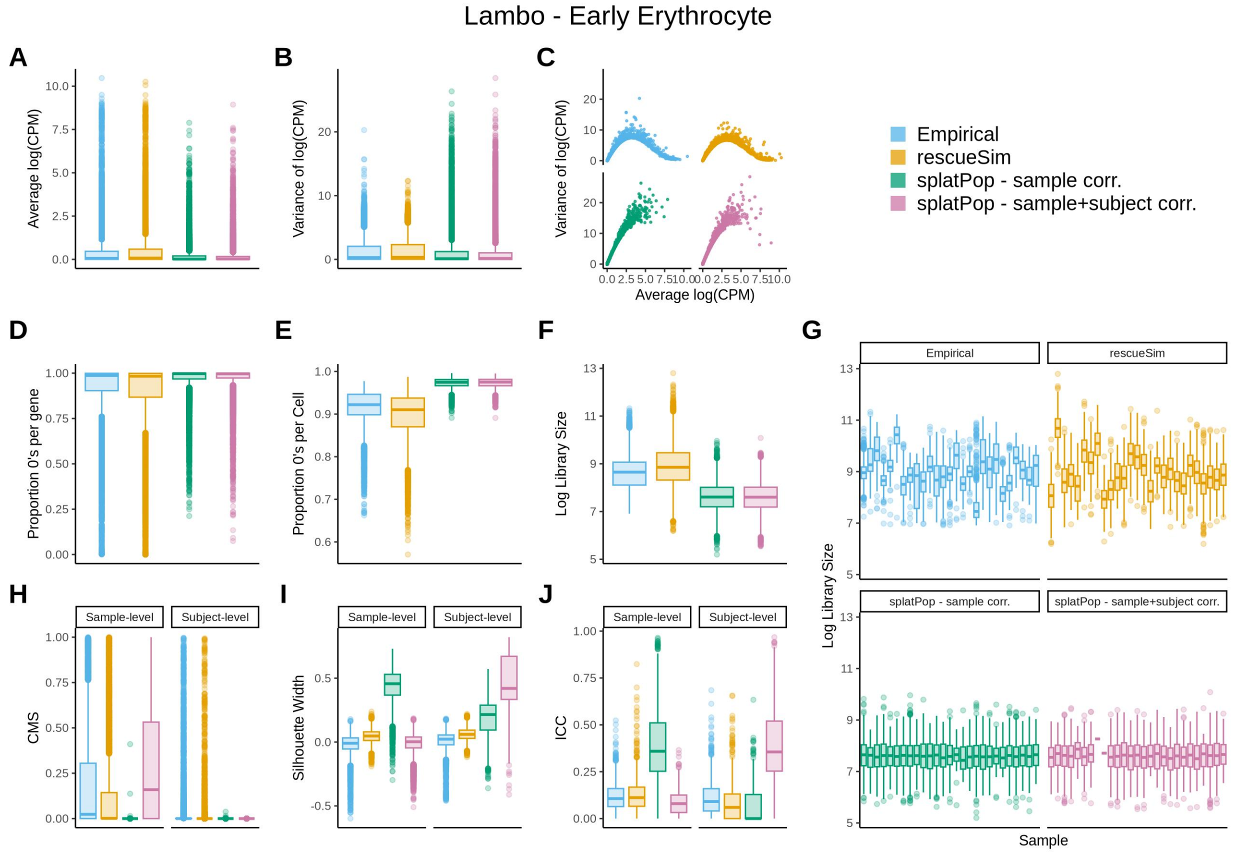

Figure S10: Comparison of key data metrics between simulated and empirical early erythrocyte cell data (Lambo et al., 2023). (A) Average log-transformed counts per million (CPM), (B) variance of log-transformed counts per million, (C) average log counts per million vs. variance of log counts per million, (D) proportion of zero counts per gene, (E) proportion of zero counts per cell (F) log-transformed library size (total counts per cell), (G) distribution of log library sizes across samples for RAM cells, (H) Cell mixing score (CMS), (I) silhouette width, and (J) intraclass correlation coefficient (ICC).

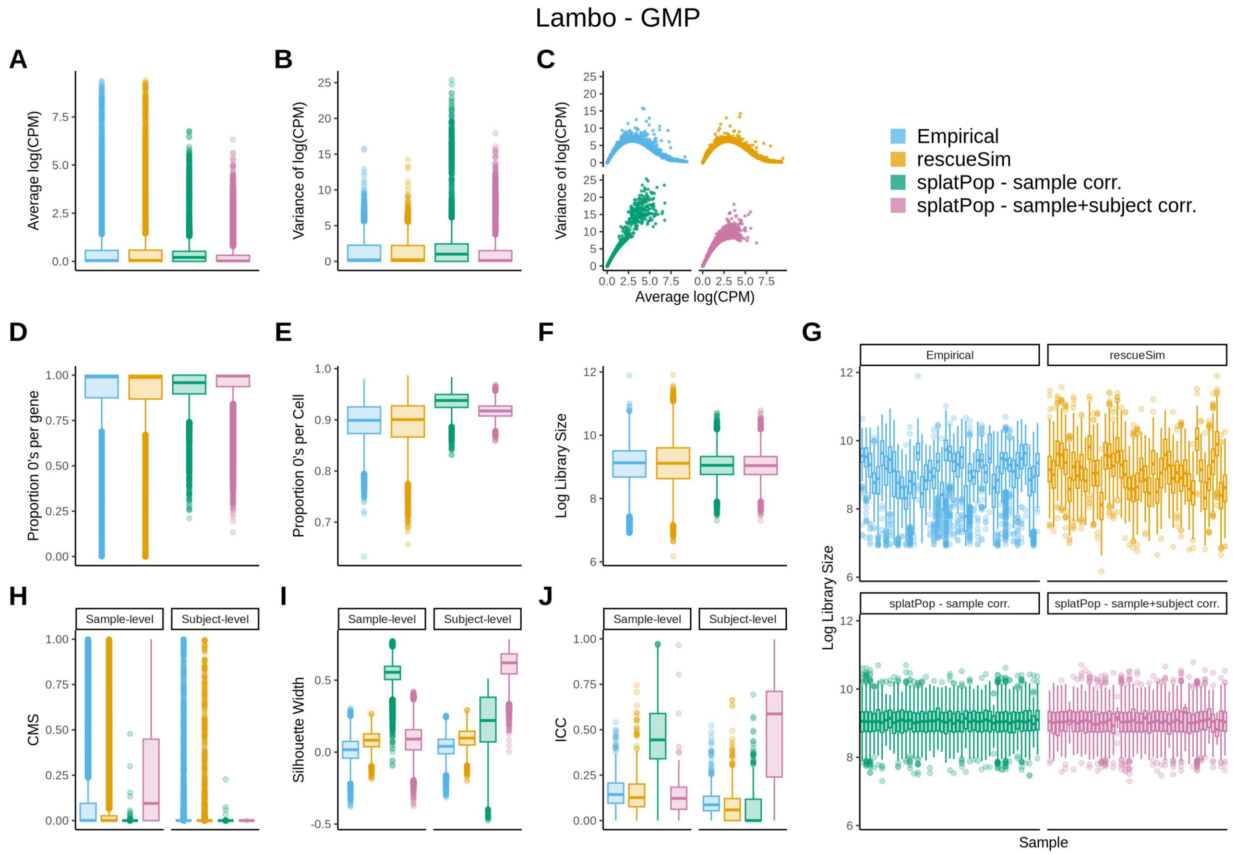

Figure S11: Comparison of key data metrics between simulated and empirical GMP cell data (Lambo et al., 2023). (A) Average log-transformed counts per million (CPM), (B) variance of log-transformed counts per million, (C) average log counts per million vs. variance of log counts per million, (D) proportion of zero counts per gene, (E) proportion of zero counts per cell (F) log-transformed library size (total counts per cell), (G) distribution of log library sizes across samples for RAM cells, (H) Cell mixing score (CMS), (I) silhouette width, and (J) intraclass correlation coefficient (ICC).

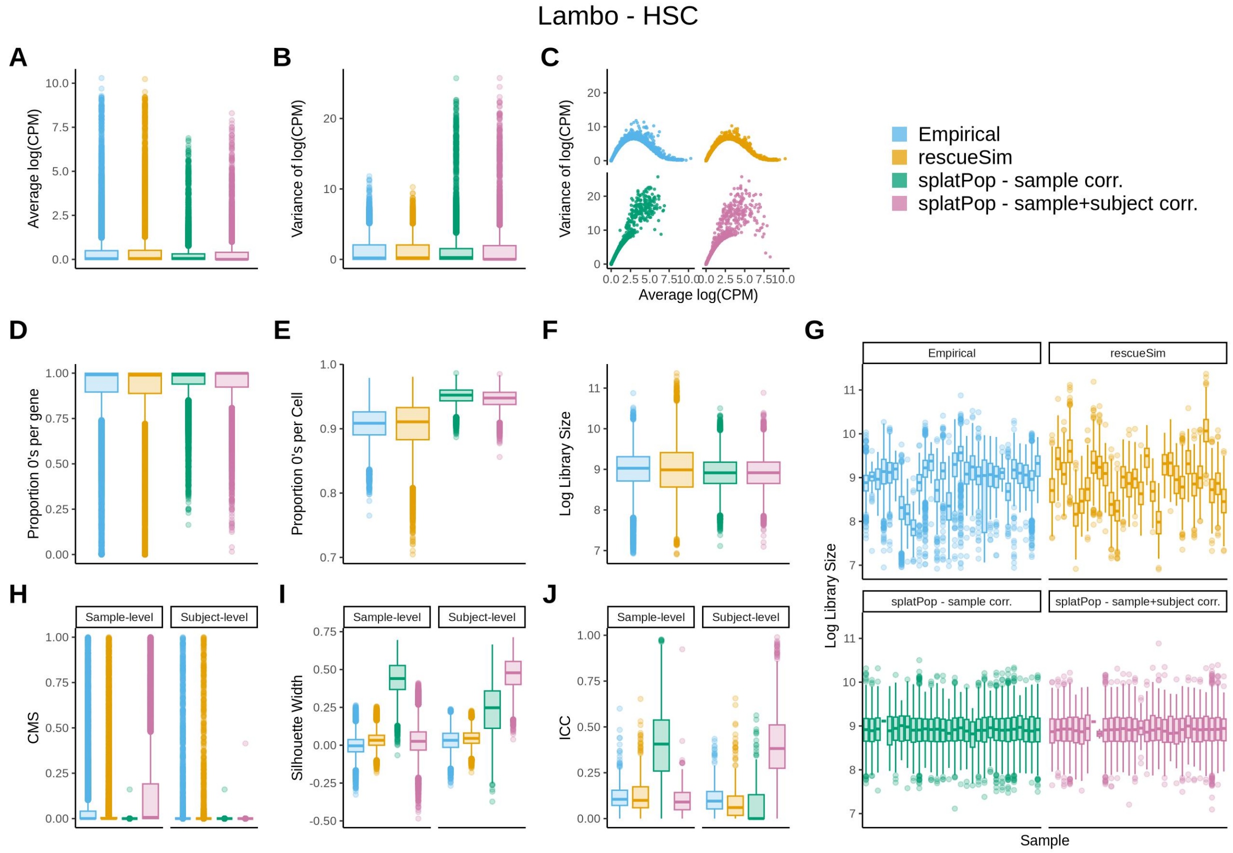

Figure S12: Comparison of key data metrics between simulated and empirical HSC cell data (Lambo et al., 2023). (A) Average log-transformed counts per million (CPM), (B) variance of log-transformed counts per million, (C) average log counts per million vs. variance of log counts per million, (D) proportion of zero counts per gene, (E) proportion of zero counts per cell (F) log-transformed library size (total counts per cell), (G) distribution of log library sizes across samples for RAM cells, (H) Cell mixing score (CMS), (I) silhouette width, and (J) intraclass correlation coefficient (ICC).

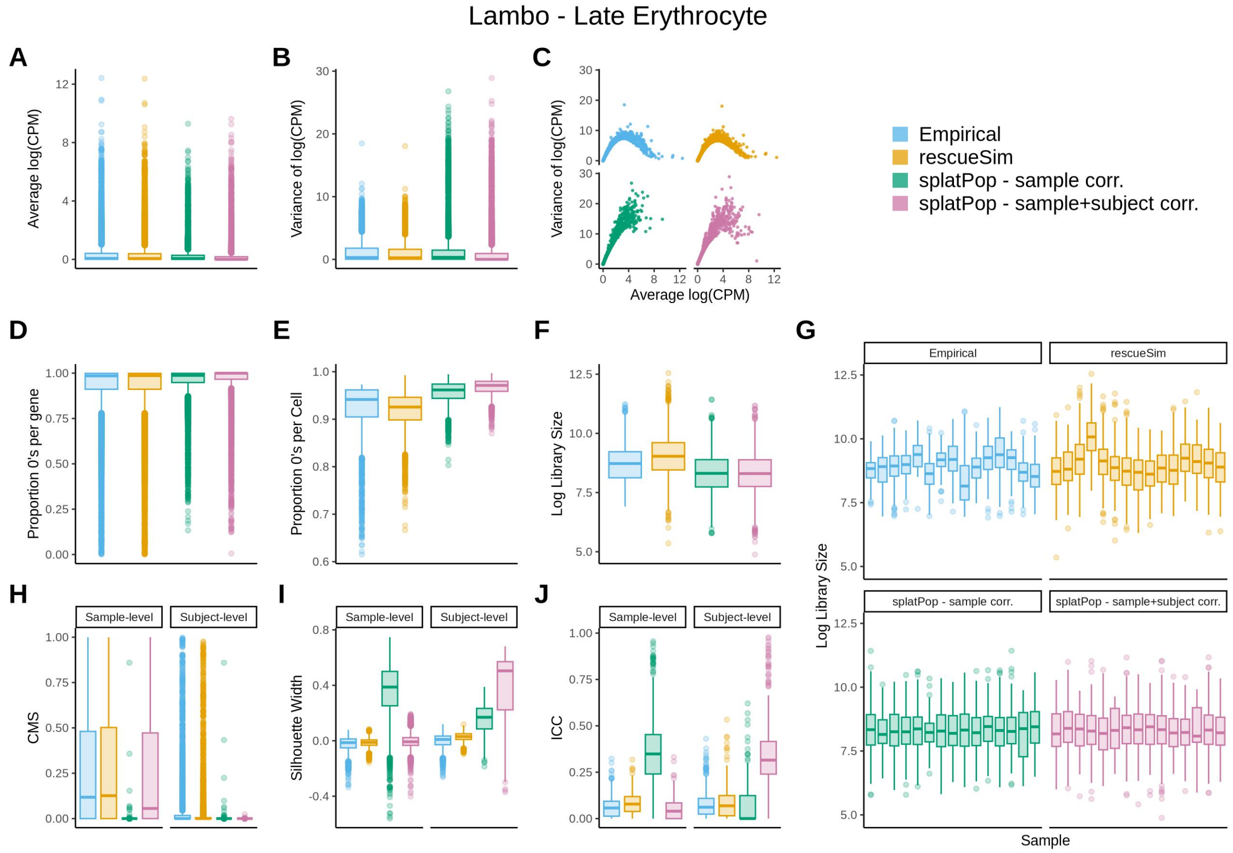

Figure S13: Comparison of key data metrics between simulated and empirical late erythrocyte cell data (Lambo et al., 2023). (A) Average log-transformed counts per million (CPM), (B) variance of log-transformed counts per million, (C) average log counts per million vs. variance of log counts per million, (D) proportion of zero counts per gene, (E) proportion of zero counts per cell (F) log-transformed library size (total counts per cell), (G) distribution of log library sizes across samples for RAM cells, (H) Cell mixing score (CMS), (I) silhouette width, and (J) intraclass correlation coefficient (ICC).

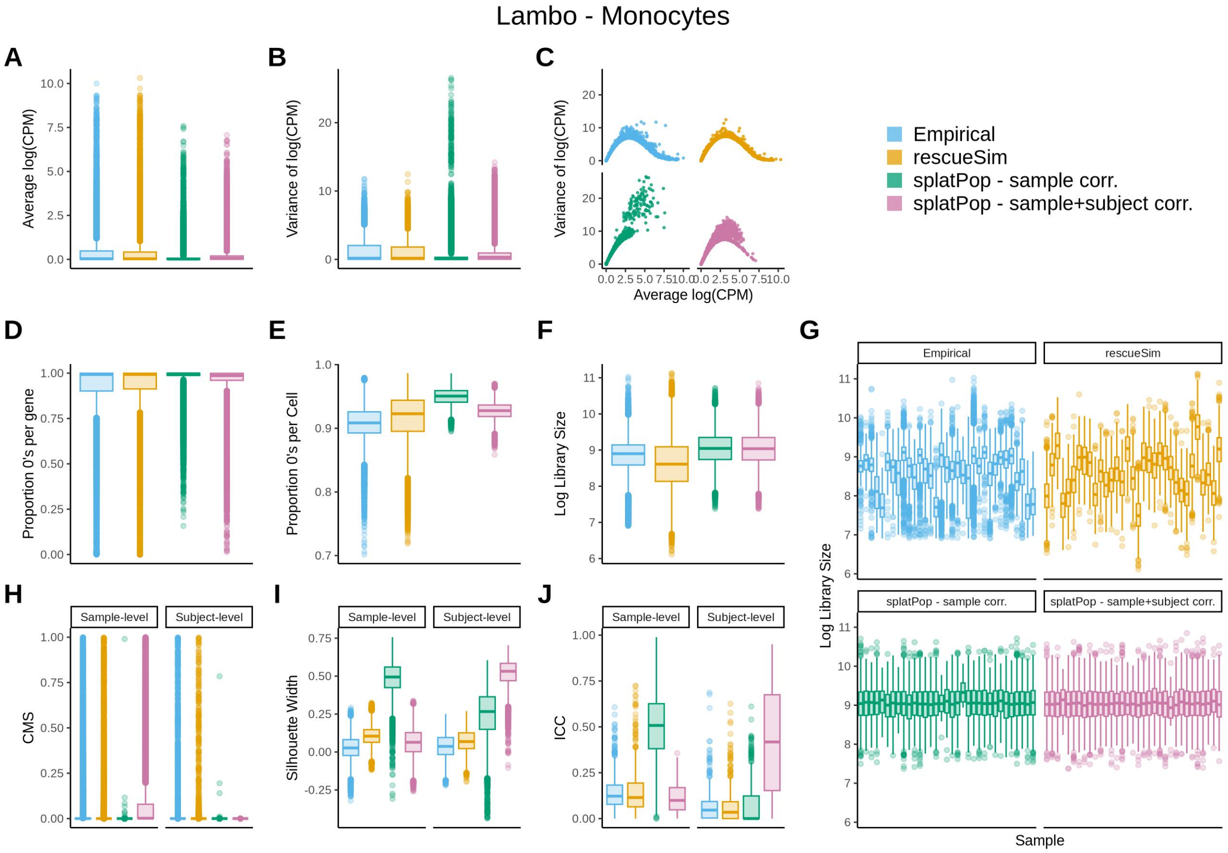

Figure S14: Comparison of key data metrics between simulated and empirical monocyte cell data (Lambo et al., 2023). (A) Average log-transformed counts per million (CPM), (B) variance of log-transformed counts per million, (C) average log counts per million vs. variance of log counts per million, (D) proportion of zero counts per gene, (E) proportion of zero counts per cell (F) log-transformed library size (total counts per cell), (G) distribution of log library sizes across samples for RAM cells, (H) Cell mixing score (CMS), (I) silhouette width, and (J) intraclass correlation coefficient (ICC).

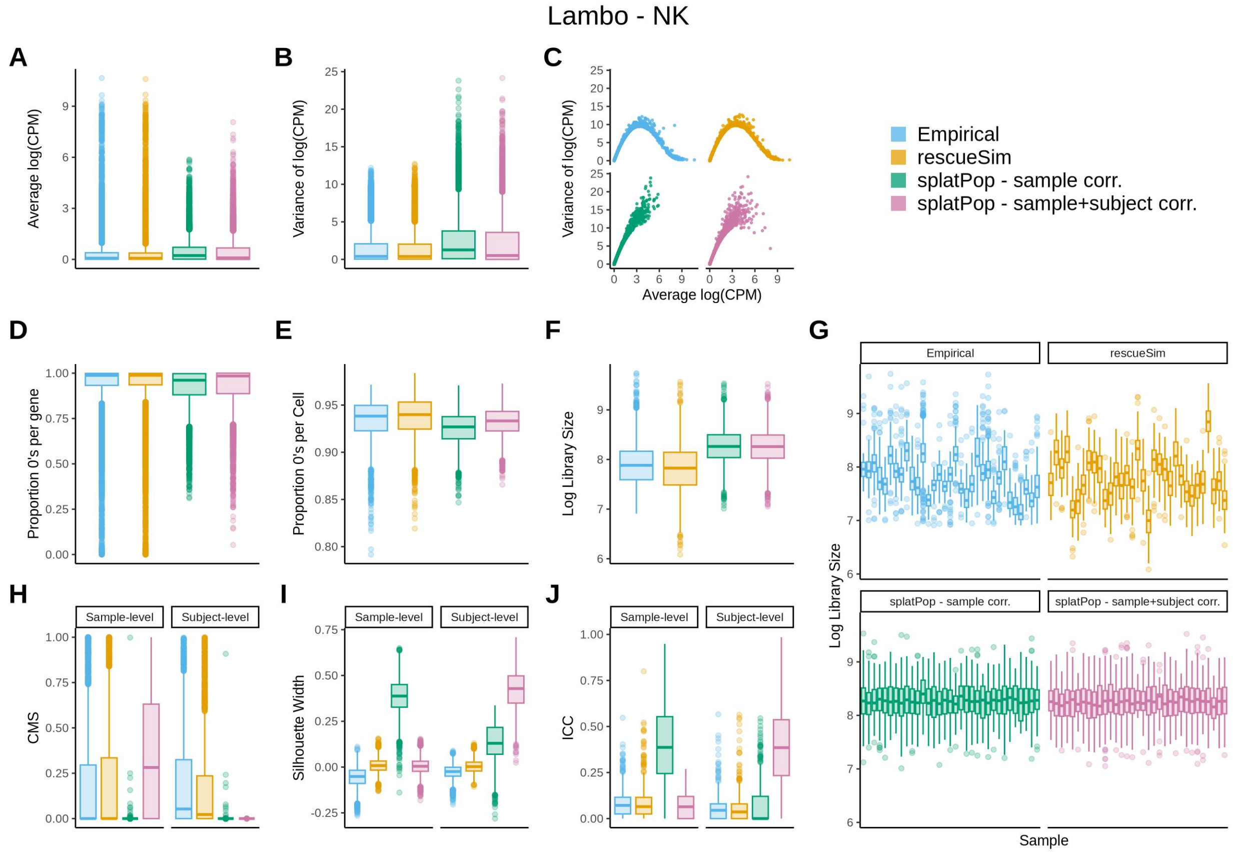

Figure S15: Comparison of key data metrics between simulated and empirical NK cell data (Lambo et al., 2023). (A) Average log-transformed counts per million (CPM), (B) variance of log-transformed counts per million, (C) average log counts per million vs. variance of log counts per million, (D) proportion of zero counts per gene, (E) proportion of zero counts per cell (F) log-transformed library size (total counts per cell), (G) distribution of log library sizes across samples for RAM cells, (H) Cell mixing score (CMS), (I) silhouette width, and (J) intraclass correlation coefficient (ICC).

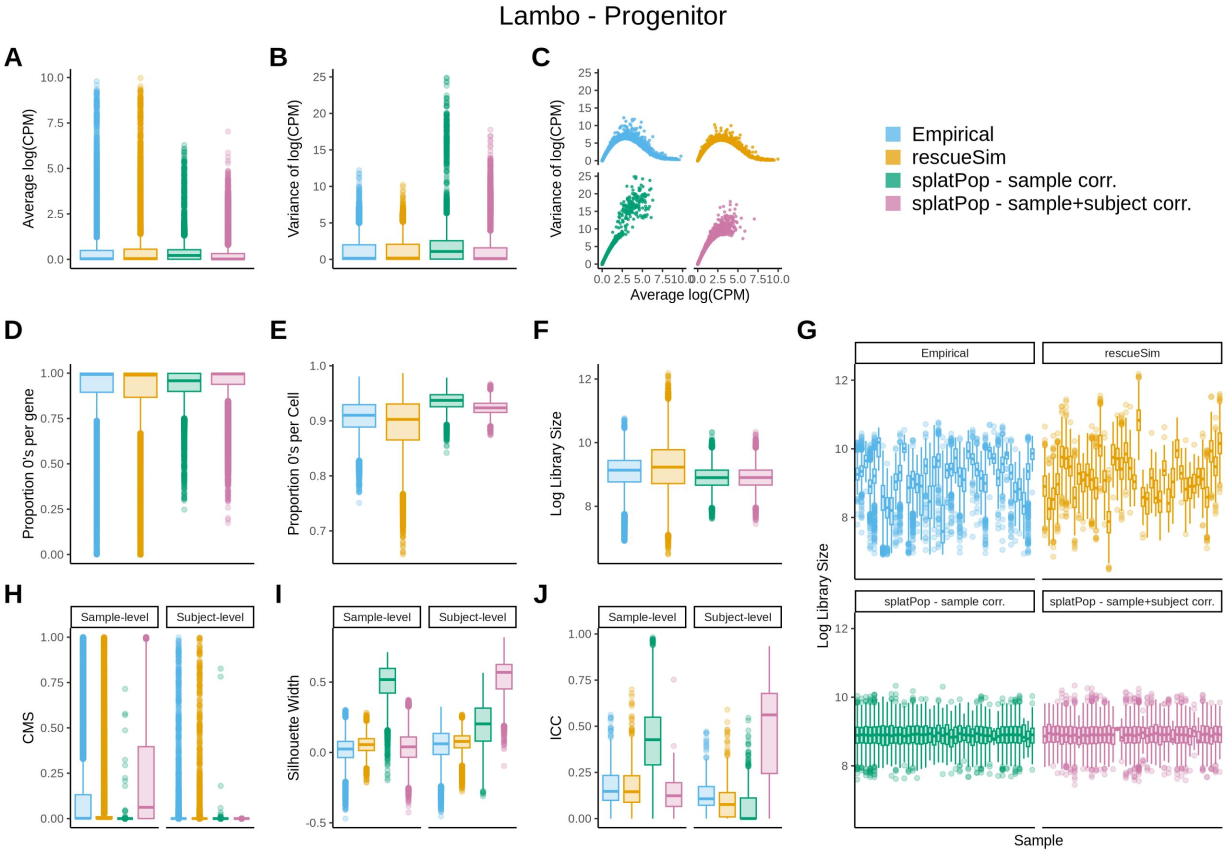

Figure S16: Comparison of key data metrics between simulated and empirical progenitor cell data (Lambo et al., 2023). (A) Average log-transformed counts per million (CPM), (B) variance of log-transformed counts per million, (C) average log counts per million vs. variance of log counts per million, (D) proportion of zero counts per gene, (E) proportion of zero counts per cell (F) log-transformed library size (total counts per cell), (G) distribution of log library sizes across samples for RAM cells, (H) Cell mixing score (CMS), (I) silhouette width, and (J) intraclass correlation coefficient (ICC).

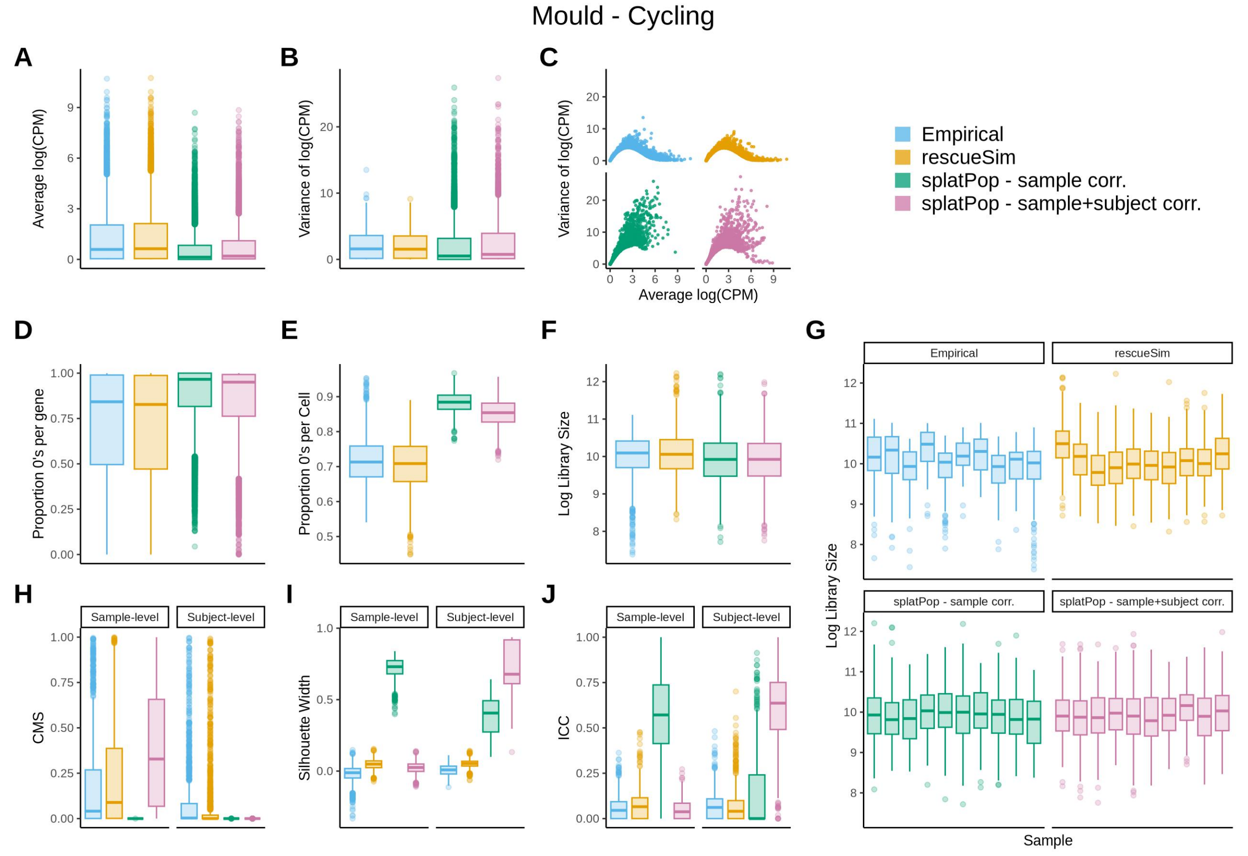

Figure S17: Comparison of key data metrics between simulated and empirical cycling cell data (Mould et al., 2020). (A) Average log-transformed counts per million (CPM), (B) variance of log-transformed counts per million, (C) average log counts per million vs. variance of log counts per million, (D) proportion of zero counts per gene, (E) proportion of zero counts per cell (F) log-transformed library size (total counts per cell), (G) distribution of log library sizes across samples for RAM cells, (H) Cell mixing score (CMS), (I) silhouette width, and (J) intraclass correlation coefficient (ICC).

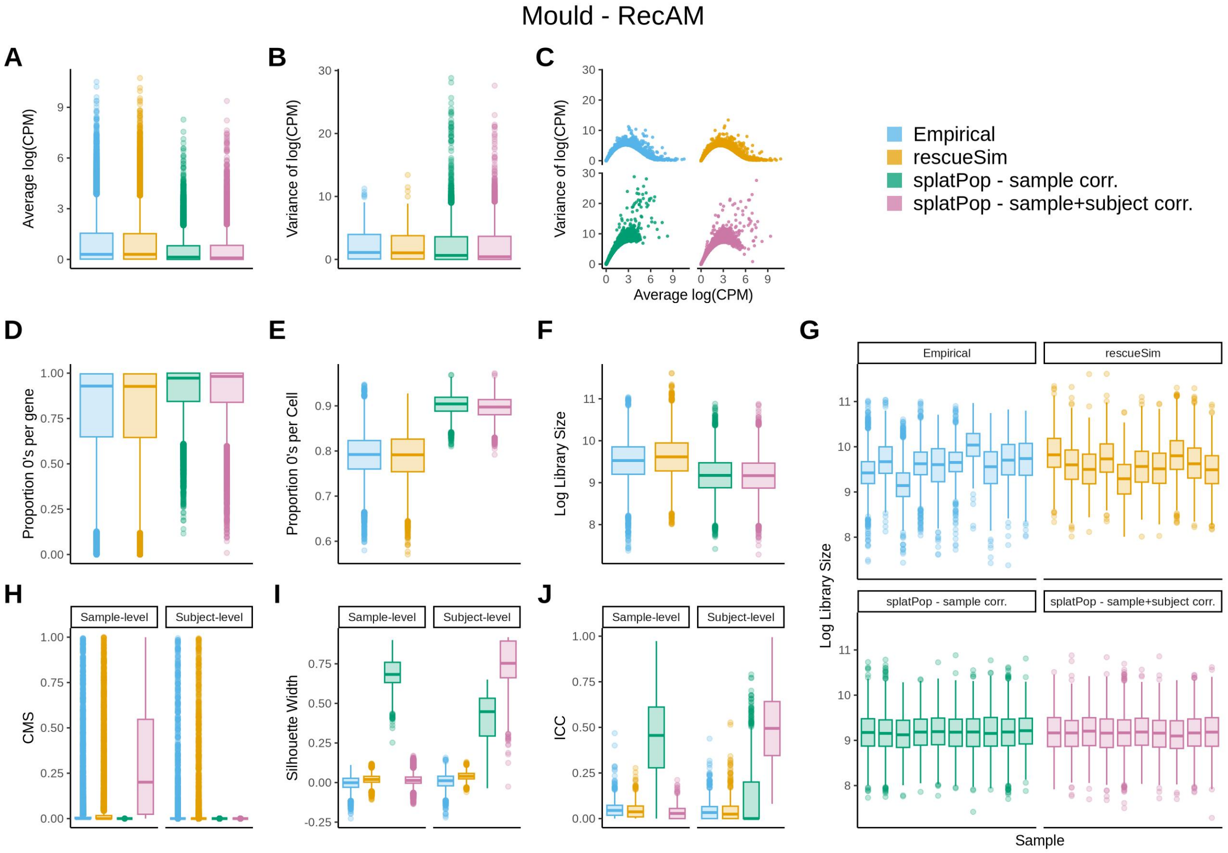

Figure S18: Comparison of key data metrics between simulated and empirical recruited macrophage (RecAM) cell data (Mould et al., 2020). (A) Average log-transformed counts per million (CPM), (B) variance of log-transformed counts per million, (C) average log counts per million vs. variance of log counts per million, (D) proportion of zero counts per gene, (E) proportion of zero counts per cell (F) log-transformed library size (total counts per cell), (G) distribution of log library sizes across samples for RAM cells, (H) Cell mixing score (CMS), (I) silhouette width, and (J) intraclass correlation coefficient (ICC).

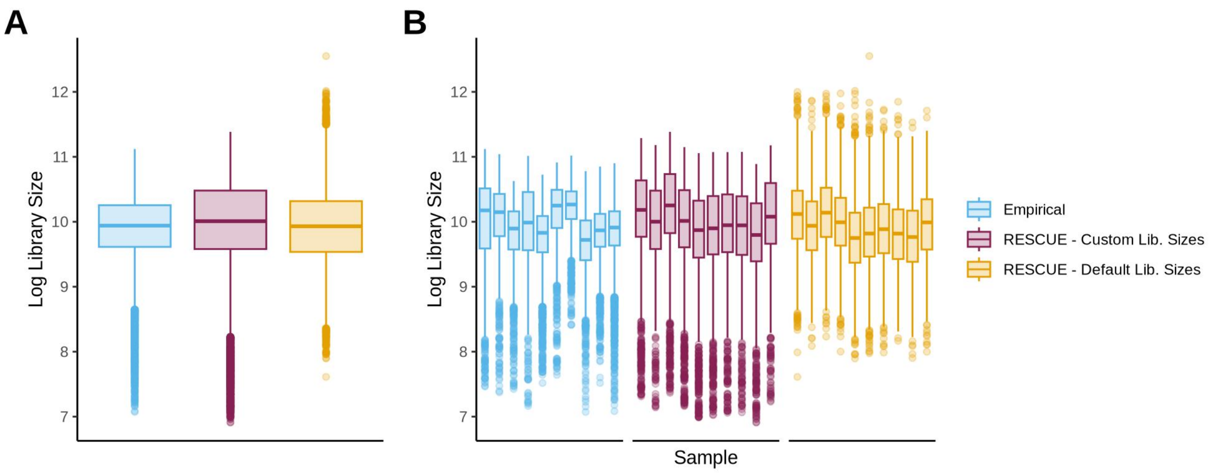

Figure S19: Comparison of library size distributions across empirical data from the RAM cells (Mould et al., 2020), rescueSim with default library sizes (drawn from a log-normal distribution), and rescueSim with custom library sizes. (A) Boxplots of log-transformed library size distributions for each group. (B) Boxplots of log-transformed library sizes split by sample for each group. For the custom library size simulation, we selected a sample from the empirical dataset whose average library size was closest to the overall dataset average and used its empirical library size distribution as input. rescueSim applies sample-specific shifts even with custom library sizes to preserve sample-to-sample variation.

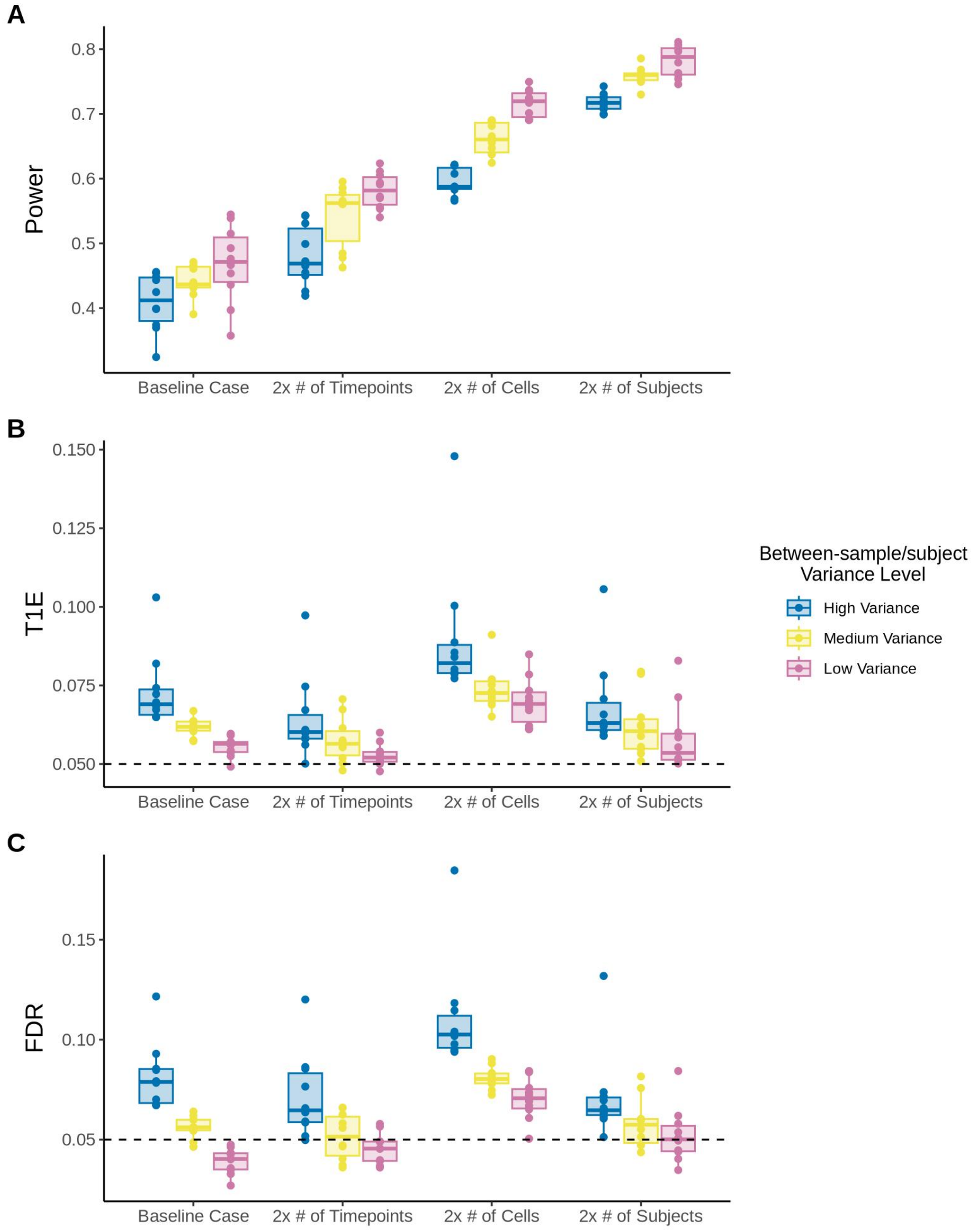

Figure S20: Comparison of metrics across simulation scenarios with varying levels of between-sample and between-subject variance in a hypothetical scRNA-seq power analysis. (A) Power, (B) Type 1 error (T1E), and (C) false discovery rate (FDR) across 10 simulations for each scenario at a 0.05 significance threshold. Between-sample between-subject variance were controlled by adjusting the parameters controlling sample- and subject-level variability in the simulation. The low variance scenario reflects reduced sample/subject-level clustering and lower intraclass correlation (ICC), while the high variance scenario reflects stronger clustering and higher ICC.

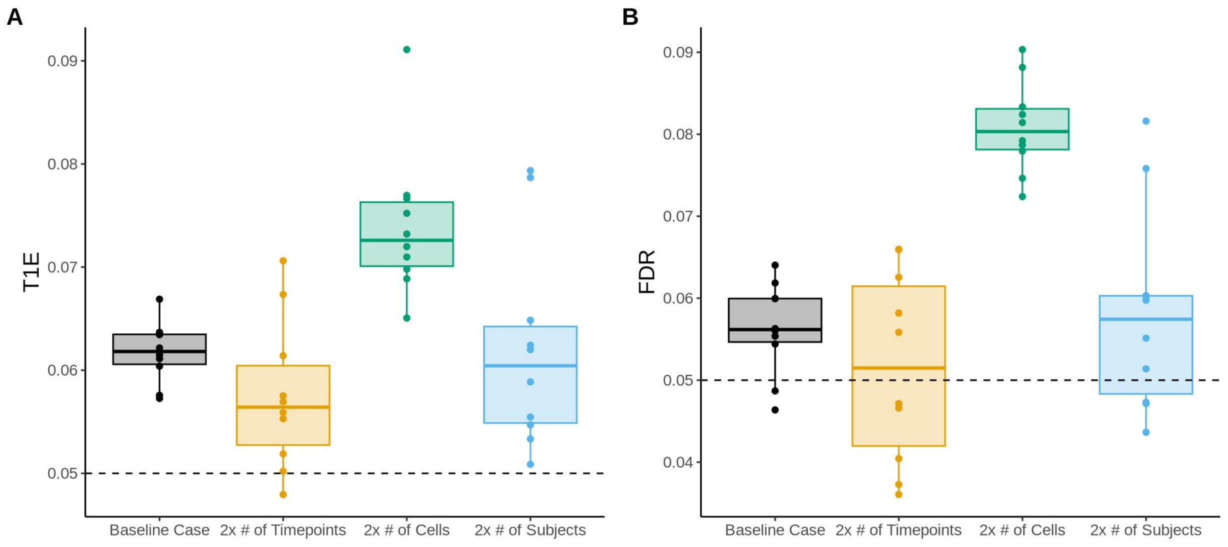

Figure S21: Comparison of error metrics across simulation scenarios in hypothetical scRNA-seq power analysis.(A) Type one error (T1E), and (B) false discovery rate (FDR) across 10 simulations for each scenario at a 0.05 significance threshold.

References

Marie Laure Delignette-Muller and Christophe Dutang. fitdistrplus: An R Package for Fitting Distributions. *Journal of Statistical Software*, 64(4):1–34, 3 2015. ISSN 1548-7660. doi: 10.18637/JSS.V064.I04. URL https://www.jstatsoft.org/index.php/jss/article/view/v064i04.

Yuhan Hao, Tim Stuart, Madeline H Kowalski, Saket Choudhary, Paul Hoffman, Austin Hartman, Avi Srivastava, Gesmira Molla, Shaista Madad, Carlos Fernandez-Granda, and Rahul Satija. Dictionary learning for integrative, multimodal and scalable single-cell analysis. *Nature Biotechnology*, 42(2):293–304, 2024. ISSN 15461696. doi: 10.1038/s41587-023-01767-y. URL https://doi.org/10.1038/s41587-023-01767-y.

Almut Lütge, Joanna Zyprych-Walczak, Urszula Brykczynska Kunzmann, Helena L. Crowell, Daniela Calini, Dheeraj Malhotra, Charlotte Soneson, and Mark D. Robinson. CellMixS: quantifying and visualizing batch effects in single-cell RNA-seq data. *Life Science Alliance*, 4(6), 6 2021. ISSN 2575-1077. doi: 10.26508/LSA.202001004. URL https://www.life-science-alliance.org/content/4/6/e202001004 https://www.life-science-alliance.org/content/4/6/e202001004.abstract.

Martin Maechler; Peter Rousseeuw; Anja Struyf; Mia Hubert; Kurt Hornik. cluster: Cluster Analysis Basics and Extensions, 2023.

Christoph Hafemeister and Rahul Satija. Normalization and variance stabilization of single-cell RNA-seq data using regularized negative binomial regression. *Genome Biology*, 20(1):1–15, 12 2019. ISSN 1474760X. doi: 10.1186/s13059-019-1874-1. URL https://link.springer.com/articles/10.1186/s13059-019-1874-1 https://link.springer.com/article/10.1186/s13059-019-1874-1.

Brian E. Vestal, Elizabeth Wynn, and Camille M. Moore. lmerSeq: an R package for analyzing transformed RNA-Seq data with linear mixed effects models. *BMC Bioinformatics*, 23(1):1–13, 12 2022. ISSN 14712105. doi: 10.1186/s12859-022-05019-9. URL https://bmcbioinformatics.biomedcentral.com/articles/10.1186/s12859-022-05019-9.
